# Aggregation of magnetic nanoparticles functionalized with *trans*-resveratrol in aqueous solution

**DOI:** 10.1186/s11671-023-03805-9

**Published:** 2023-04-19

**Authors:** Thi-Nga Nguyen, Quang-Hung Tran, Ferial Terki, Clarence Charnay, Xavier Dumail, Corine Reibel, Guillaume Cazals, Gilles Valette, Christian Jay-Allemand, Luc P. R. Bidel

**Affiliations:** 1 UMR IATE, Institut Agro, INRAE, University of Montpellier, 34060 Montpellier, France; 2 PhyMedExp UMR CNRS 9214 – Inserm U1046, 34295 Montpellier Cedex 05, France; 3 Institute of Natural Products Chemistry, Vietnam Academy of Science and Technology, 18 Hoang Quoc Viet, Hanoi, 100000 Vietnam; 4 eV-Technologies, 2 Esplanade Anton Philips, Bâtiment 5, 14460 Colombelles, France; 5 Institut Charles Gerhardt UMR 5253 CNRS-UM, Université de Montpellier, 34095 Montpellier, France; 6 IBMM UMR5247, CNRS, ENSCM, Université de Montpellier, Place Eugène Bataillon, 34095 Montpellier Cedex 5, France

**Keywords:** Nanoparticle aggregation, *trans*-resveratrol, Magnetic nanoparticles, Mesoporous silica shells, Nanoparticle functionalization

## Abstract

In the framework of a protein–ligand-fishing strategy to identify proteins that bind to *trans-*resveratrol, a natural phenolic compound with pharmacological benefits, we have developed magnetic nanoparticles covalently linked to *trans*-resveratrol through three different derivatives and examined their aggregation behavior in aqueous solution. The monodispersed magnetic core (18 nm diameter) with its mesoporous silica shell (93 nm diameter) exhibited a notable superparamagnetic behavior useful for magnetic bioseparation. The hydrodynamic diameter, deduced from dynamic light scattering analysis, of the nanoparticle increased from 100 to 800 nm when the aqueous buffer changed from pH 10.0–3.0. A size polydispersion occurred from pH 7.0–3.0. In parallel, the value of the extinction cross section increased according to a negative power law of the UV wavelength. This was mainly due to light scattering by mesoporous silica, whereas the absorbance cross section remained very low in the 230–400 nm domain. The three types of resveratrol-grafted magnetic nanoparticles exhibited similar scattering properties, but their absorbance spectrum was consistent with the presence of *trans*-resveratrol. Their functionalization increased their negative zeta potential when pH increased from 3.0 to 10.0. The mesoporous nanoparticles were monodispersed in alkaline conditions, where their anionic surface strongly repulsed each other but aggregated progressively under van der Waals forces and hydrogen bonding when negative zeta potential decreased. The characterized results of nanoparticle behavior in aqueous solution provide critical insight for further study of nanoparticles with proteins in biological environment.

**Supplementary Information:**

The online version contains supplementary material available at 10.1186/s11671-023-03805-9.

## Introduction

Phenolic compounds interact with many peptides and proteins and modulate metabolism [1], signaling pathways [2, 3], and reactions of organisms to environmental cues and bio-aggressors [4]. Among phenolic compounds, *trans-*resveratrol (3,5,4′-trihydroxystilbene) is the main monomer of the stilbenoid subclass. Produced by plant species, it is considered with an increasing interest in many biological fields related to biocontrol [5], cosmetic [6, 7], nutrition and medicine [8–10]. Among the 11,987 interactions between 369 polyphenols and 5699 unique proteins annotated in the STITCH interactome database, *trans*-resveratrol is referenced in 738 interactions at the 4^th^ rank of the highest interacting phenolic compound [1]. Identification of new peptide and protein targets of *trans-*resveratrol is an essential step to elucidate their mechanism of action as fungistatic and fungicide [11, 12], bacteriostatic and bactericide [13], antioxidant, and many healthy properties reviewed [14]: vasorelaxant agent, anti-hypertensive and cardioprotective agent, estrogeno-mimetic agent, anti-diabetic agent, peptide anti-aggregative agent, and neuroprotective agent, anti-inflammatory agent, anti-angiogenic agent, immunomodulatory agent, antitumor drug, radioprotective agent. To improve the bioavailability of this poorly soluble *trans-*resveratrol, many efforts have been carried out for vectorizing it into various vehicles reviewed such as vesicular systems (nanoemulsion, nanomicelles, cyclodextrins complexes) [15–17], and nanocarriers (solid lipid nanoparticles, nanosuspensions, nanocapsules, protein-based or chitosan-based nanoparticles) [18]. Recently, mesoporous silica nanoparticles have been used as a nanocarrier of adsorbed *trans-*resveratrol for active targeted delivery with progressive release [19–21]. Furthermore, magnetic iron oxide nanoparticles functionalized by an organosilane coupling agent (3-chloropropyltriethoxysilane, CPTES) have also been developed [22].

Nanoparticle designing for protein–ligand fishing remains challenging. Among the diversity of magnetic nanoparticle available (iron, nickel, cobalt), some iron oxide nanoparticles exhibit superparamagnetic property in the temperature range of living cells when they are submitted to an external magnetic field, without remanence left when the field is removed. Compared to ferromagnetic permanent nano-magnet, they exhibit higher magnetic susceptibility and lower aggregation tendency. They are more suitable to monitor protein–ligand fishing [23]. Parameters such as their size, shape, coating, functionalization, and zeta potential can have a great impact on their diffusion, uptake capacity, toxicity, and affinity for their target, their colloidal stability in aqueous proteins necessary for preserving native proteins. The aggregation behavior in aqueous condition appears as a critical phase to determine how to use it. Although there are researches on the aggregation of iron oxide nanoparticle [24, 25], the functionalized magnetic nanoparticle is rarely documented. Indeed, the properties of functionalized nanoparticles should be carefully characterized to avoid any undesirable activity and further understanding for later uses [26–28].

Here, we report the impact of pH to the aggregation of magnetic nanoparticle grafted with *trans*-resveratrol. Initially, we present the synthesis and physical characterizations of superparamagnetic iron oxide core with a high magnetic moment, the synthesis of its mesoporous silica shell, the synthesis of three silanized resveratrol derivatives, and their grafting on silica shell. To examine *trans-*resveratrol covalent grafting on magnetic nanoparticles (MNPs), FT-IR, and UV–Vis were used. We further analyze the impact of pH on the aggregation of the three resveratrol derivatives grafted MNPs (**CS1**, **CS2**, and **CS3**). The nanoparticles (MNPs) specifically designed in this work will allow future studies of *trans*-resveratrol interactions with proteins using a magnetic sensor. Magnetic cores of 18 nm diameter have been coated with a silica shell of 93 nm diameter to avoid their oxidation, to decrease their density and increase their buoyancy, and to provide a better colloidal behavior due to the interactions of silanols with water.

This MNPs-resveratrol system is the first result to develop new studies toward quantification of grafting ligand on nanoparticles and screening of specific proteins for *trans*-resveratrol by ligand fishing.

## Materials and methods

### Synthesis of magnetic core

Nanoparticles were synthesized by the decomposition method [29]. The flask contains 5.055 g *n*-docosane (99%, Acros organic), 0.181 g iron oxide hydrate (catalyst grade, 30–50 Mesh, Sigma-Aldrich), and 3 g oleic acid (> 99%, Sigma-Aldrich). The mixture was put under vacuum for 30 min, then heated under reflux to 340 °C under argon gas for 1h30m. The mixture was then dispersed in *n*-pentane (for analysis RPE) and diethyl ether/ethanol (2:1). Nanoparticles were collected by centrifugation and washed with diethyl ether/ethanol 2:1. Finally, all nanoparticles were dissolved in 15 mL CHCl_3_ (RPE for analysis) and 200 µL oleylamine, then stored at 4 °C.

### Enwrapping of a magnetic core with a mesoporous silica shell

The formation of the silica shell was carried out with tetraethylorthosilicate (TEOS) agent [30]. The flask containing 0.125 g cetyltrimethylammonium bromide (CTAB) (≥ 98%, Sigma-Aldrich), 60 mL pure water (for HPLC, Sigma-Aldrich), and 440 µL NaOH 2 M was stirred at 70 °C for 1 h. The iron oxide core in CHCl_3_ (11 × 100 µL) was then added at 3–4 min intervals. The mixture was stirred at 80 °C for 1h30 before the addition of 100 µL of TEOS (≥ 99% (GC), Sigma-Aldrich), followed by another addition of 500 µL TEOS 30 min later. The mixture was kept at 80 °C for another 1h30 period. Nanoparticles were collected by centrifugation at 20 rpm for 15 min (Allegra64R centrifuge, Beckman coulter, USA), then suspended in 0.075 M NH_4_NO_3_ for one night. Nanoparticles were washed with absolute ethanol (99%, VWR) and pure water before being dried under vacuum.

Each batch of nanoparticles was examined using transmission electronic microscopy (TEM) and morphometric measurements were carried out with magnification at 15,000× and 50,000× using a JEOL 1200 EXII microscope (Japan). Magnetic data were collected with a Quantum Design MPMS-XL SQUID magnetometer working in the temperature range 5–300 K.

### Silanization of *trans*-resveratrol

**Scheme 1:**
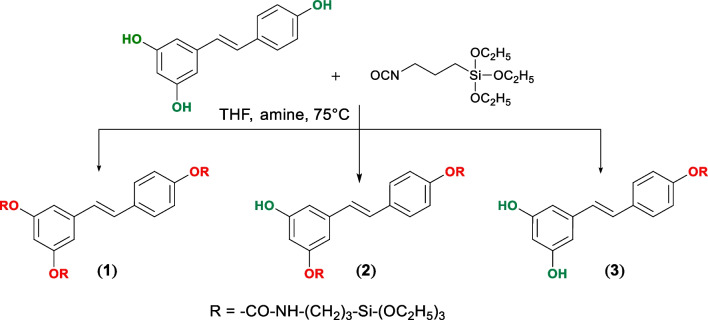
The synthesis pathway of *trans*-resveratrol derivatives **(1), (2)** and** (3)**

To graft *trans*-resveratrol on the surface of core/shell nanoparticle, silane-*trans*-resveratrol derivatives were synthesized by the base-catalyzed reaction of (triethoxysilyl)propyl isocyanate with *trans*-resveratrol in tetrahydrofuran THF shown in Scheme 1.

1.14 g *trans*-resveratrol (5 mmol, > 99%, TCI Europe) and 2.50 mL 3-(triethoxysilyl)propyl isocyanate (10 mmol, 95%, Sigma) were dissolved in 20 mL dry THF, then 70 µL *N*,*N*-diisopropylethylamine (> 99%, TCI Europe) was added. The reaction was stirred under reflux at 70 °C for 3 days. The reaction was followed by infrared spectroscopy and thin layer chromatography (TLC) using pre-coated TLC sheets with UV fluorescent silica gel (Merck 60F254). The solvent was then removed under reduced pressure and the mixture of (**1**), (**2**), and (**3**) was purified by flash chromatography (Isolera One, Biotage, Sweden) using a Biotage SNAP ULTRA column and *n*-hexane/acetone 2:1 as eluent, to give **1** (50%), **2** (30%), **3** (16%).

To elucidate the structures of **1**, **2**, and **3**, NMR spectra were measured in DMSO-*d*_*6*_, using a Bruker Avance 400 MHz spectrometer. Chemical shifts (δ) were expressed in ppm relative to tetramethyl silane (TMS) and coupling constants (*J*) in Hz. Mass spectra were obtained using a Synapt G2-S high-definition mass spectrometry system (Waters Corp., Milford, MA) equipped with an electrospray ionization (ESI) source. Since compound **1** did not ionize well in the ESI source, its mass spectrum was obtained using a Bruker RapifleX MALDI-TOF spectrometer (Bruker Daltonik GmbH, Bremen, Germany) equipped with a Smartbeam 3D laser. The MALDI-TOF analysis was performed in positive ion reflectron mode over a mass range of m/z 450–3000 with or without cationization agent. The matrix (DHB, 10 g L^−1^) and the sample were mixed (10:1 v/v) and 1 µL of the mixture was spotted onto the MALDI target plate and air-dried. For the experiments with a cationizing agent, 1 μL of 10 g L^−1^ sodium trifluoroacetate was added to the mixture before spotting.

^**1**^**H-NMR of 1:** δ ppm (400 MHz, DMSO-*d*_6_, Me_4_Si): 7.81 (m, 3H, N–H), 7.60 (d broad, *J* = 8 Hz, 2H, H-3′, H-5′), 7.32 (d, *J* = 16 Hz, 1H, H-α), 7.20 (m, 3H, H-β, H-2′, H-6′), 7.11 (d, *J* = 8 Hz, 2H, H-2, H-6), 6.76 (t, *J* = 2 Hz, 1H, H-4), 3.77 (q, *J* = 6 Hz, 18H, –Si–O–CH_2_–CH_3_), 3.05 (q, *J*_1_ = 4 Hz, *J*_2_ = 8 Hz, 6 H, –Si–CH_2_–CH_2_–CH_2_–N), 1.54 (dt, *J*_1_ = 4 Hz, *J*_2_ = 8 Hz, 6H, –Si–CH_2_–CH_2_–CH_2_–N), 1.17 (m, 27H, –Si–O–CH_2_–CH_3_), 0.59 (t, *J* = 6 Hz, 6H, Si–CH_2_–CH_2_–CH_2_–N). ^**13**^**C-NMR**: δ ppm (101 MHz, DMSO-*d*_6_, Me_4_Si) 154.0 (C=O), 151.7 (C-3, C-5), 150.8 (C-4′), 138.9 (C-1′), 133.5 (C-3′, C-5′), 129.1 (Cβ), 127.4 (C-α), 126.7 (C-1),, 122.0 (C-2′, C-6′),, 116.2 (C-2, C-6), 114.6 (C-4), 57.7 (Si–O–CH_2_–CH_3_), 54.9 (NH–CH_2_–CH_2_–CH_2_–Si), 22.8 (NH-CH_2_-CH_2_-CH_2_-Si), 18.2 (Si–O–CH_2_–CH_3_), 7.2 (NH–CH_2_–CH_2_–CH_2_–Si). HRMS (ESI) for C_44_H_75_N_3_O_15_Si_3_ [(M + formic acid) – H]^−^: m/z calc. 1014.4514. MALDI-TOF [M + Na] m/z 992.4.

^**1**^**H-NMR of 2:** δ ppm (400 MHz, DMSO-*d*_6_, Me_4_Si): 9.65 (s, 1H, 3-OH), 7.76 (s broad, 2H, –NH), 7.64–7.56 (m, 2H, H-3′, H-5′), 7.18–7.08 (m, H-2′, H-6′, H-α, H-β), 6.80 (m, 2H, H-2, H-6,), 6.41 (t, *J* = 2 Hz, 1H, H-4), 3.76 (t, *J* = 7 Hz, 12H, –Si–O–CH_2_–CH_3_), 3.05 (t, *J*_1_ = 7.5 Hz, *J*_2_ = 4 Hz, 4H, –Si–CH_2_–CH_2_–CH_2_–N), 1.53 (m, 4H, –Si–CH_2_–CH_2_–CH_2_–N), 1.16 (m, 18H, –Si–O–CH_2_–CH_3_), 0.59 (m, 4H, Si–CH_2_–CH_2_–CH_2_–N). ^**13**^**C-NMR**: δ ppm (101 MHz, DMSO-*d*_6_, Me_4_Si) 158.2 (C-5), 154.3 (C-3), 154.2 (C=O), 150.7 (C-4′), 138.9 (C-1′), 133.6 (C-1), 128.1 (C-β), 127.7 (C-5′), 127.3 (C-3′), 122.0 (C-α), 115.8 (C-2′), 115.6 (C-6′), 110.4 (C-2), 110.2 (C-6), 108.4 (C-4), 57.7 (Si–O–CH_2_–CH_3_), 43.2 (NH–CH_2_–CH_2_–CH_2_–Si), 23.5 (NH–CH_2_–CH_2_–CH_2_–Si), 18.1 (Si–O–CH_2_–CH_3_), 7.2 (NH–CH_2_–CH_2_–CH_2_–Si). HRMS (ESI) for C_34_H_54_N_2_O_11_Si_2_ [M–H]^−^: m/z calc. 721.3196. MALDI-TOF [M + Na + H]^+^ m/z 745.3.

^**1**^**H-NMR of 3:** δ ppm (400 MHz, DMSO-*d*_6_, Me_4_Si): 9.24 (s, 2H, 3-OH, 5-OH), 7.76 (t, *J* = 6 Hz, 1H, –NH–CO), 7.57 (t, *J* = 8 Hz, 2H, H-3′, H-5′), 7.09–7.02 (m, 4H, H-α H-β, H-2′, H-6′), 6.44 (m, 2H, H-2, H-6), 6.17 (t, *J* = 2 Hz, 1H, H-4), 3.77 (t, *J* = 7 Hz, 6H, –Si–O–CH_2_–CH_3_), 3.05 (t, *J* = 7 Hz, 2H, N–CH_2_–CH_2_–CH_2_–Si), 1.53 (q, *J* = 6 Hz, 2H, –N–CH_2_–CH_2_–CH_2_–Si), 1.18 (t, *J* = 7 Hz, 9H, –Si–O–CH_2_–CH_3_), 0.59 (t, *J* = 6 Hz, 4H, N–CH_2_–CH_2_–CH_2_–Si). ^**13**^**C-NMR**: δ ppm (101 MHz, DMSO-*d*_6_, Me_4_Si) 159.2 (C-3, C-5), 154.9 (C=O), 151.0 (C-4′), 139.4 (C-1′), 134.5 (C-1), 129.3 (C-β), 127.8 (C-3′, C-5′), 127.6 (C-α), 122.5 (C-2′, C-6′), 105.3 (C-2, C-6), 102.9 (C-4), 58.4 (Si–O–CH_2_–CH_3_), 43.8 (NH–CH_2_–CH_2_–CH_2_–Si), 23.5 (NH–CH_2_–CH_2_–CH_2_–Si), 18.8 (Si–O–CH_2_–CH_3_), 7.8 (NH–CH_2_–CH_2_–CH_2_–Si). HRMS (ESI) for C_24_H_33_NO_7_Si [M–H]^−^: m/z calc. 474.1947. MALDI-TOF [M + Na + H]^+^ m/z 498.2

### Functionalized nanoparticles preparation

Three flasks, each containing 100 mg of core/shell (**CS**) nanoparticles, were added with 1 mL toluene (ACS reagent, Sigma-Aldrich). Three other flasks contained 150 mg of **1**, **2**, and **3**, respectively, in 1 mL toluene. The 1 mL suspensions of **CS** nanoparticles were added to the latter flasks along with 60 µL of ultrapure water (18.5 MΩ) then stirred and heated up to 70 °C for 15 h. The functionalized **CS1**, **CS2**, and **CS3** nanoparticles were then collected by washing with ethanol and drying under vacuum for 3–4 h.

The successful grafting of **1**, **2**, and **3** on **CS** was verified by recording the UV and IR absorption spectra of CS**1**, **CS2**, and **CS3** using a UV-1800 spectrometer (Shimadzu, Japan) with 10 mm quartz cuvettes (101-QS, Hellma™, Germany), and a Spectrum Two FT-IR spectrometer (Perkin Elmer, England) in transmittance mode with KBr disks.

### Hydrodynamic diameter

The distribution of hydrodynamic diameter (*d*_H_) of the nanoparticles was studied at 25 °C by dynamic light scattering (DLS) using a Zetasizer Nano-ZS (Malvern Instruments Limited, UK), adapted to assess particle sizes between 5 and 1000 nm. The instrument was equipped with a 633-nm diode laser and a DTS1070 cell. DLS was analyzed at the back scattering angle of 173°. In this configuration, the contributions of rotational diffusion effects in the observed autocorrelation profiles can be neglected and the translational diffusion coefficient, D, can be assessed [31]. Five replicates of each colloidal suspension were homogenized by an ultrasound bath for 5 min and were immediately analyzed by Zetasizer Nano-ZS over 3 min.

### Zeta potential

Zeta potential was also measured with the Zetasizer Nano-ZS. An electric field of 120 V was applied across the DTS1060C zeta cell and the electrophoretic mobility of the colloidal suspension was then measured by laser Doppler velocimetry (LDV). The zeta potential is referred to as the electrostatic charge at the splining plane boundary separating ions within the diffuse layer which moves with the nanoparticles, and ions that remain with the bulk water, corresponding to the Stern shell. Zeta potential is related to the surface electrostatic charge of nanoparticles [32].

### Spectrophotometric analyses

For extinction cross section determination, all nanoparticles were initially suspended in absolute ethanol and were then diluted at the final suspension concentration of 50 ppm (50 µg/mL) in aqueous tris(hydroxymethyl)aminomethane (TRIS) buffer (50 mM), previously adjusted to pH 3.0, 5.0, 7.0, and pH 10.0 with HCl 37% and HCl 0.1 N. Extinction was recorded by EnSpire microplate reader (PerkinElmer, Singapore) at 1 nm step in front face configuration using 96 wells black microplates (UV-Star µClear, Greiner-Bio-One, Germany) set up at 27 °C.

## Results

### Characterization of magnetic core and core/shell nanoparticles

**Fig. 1:**
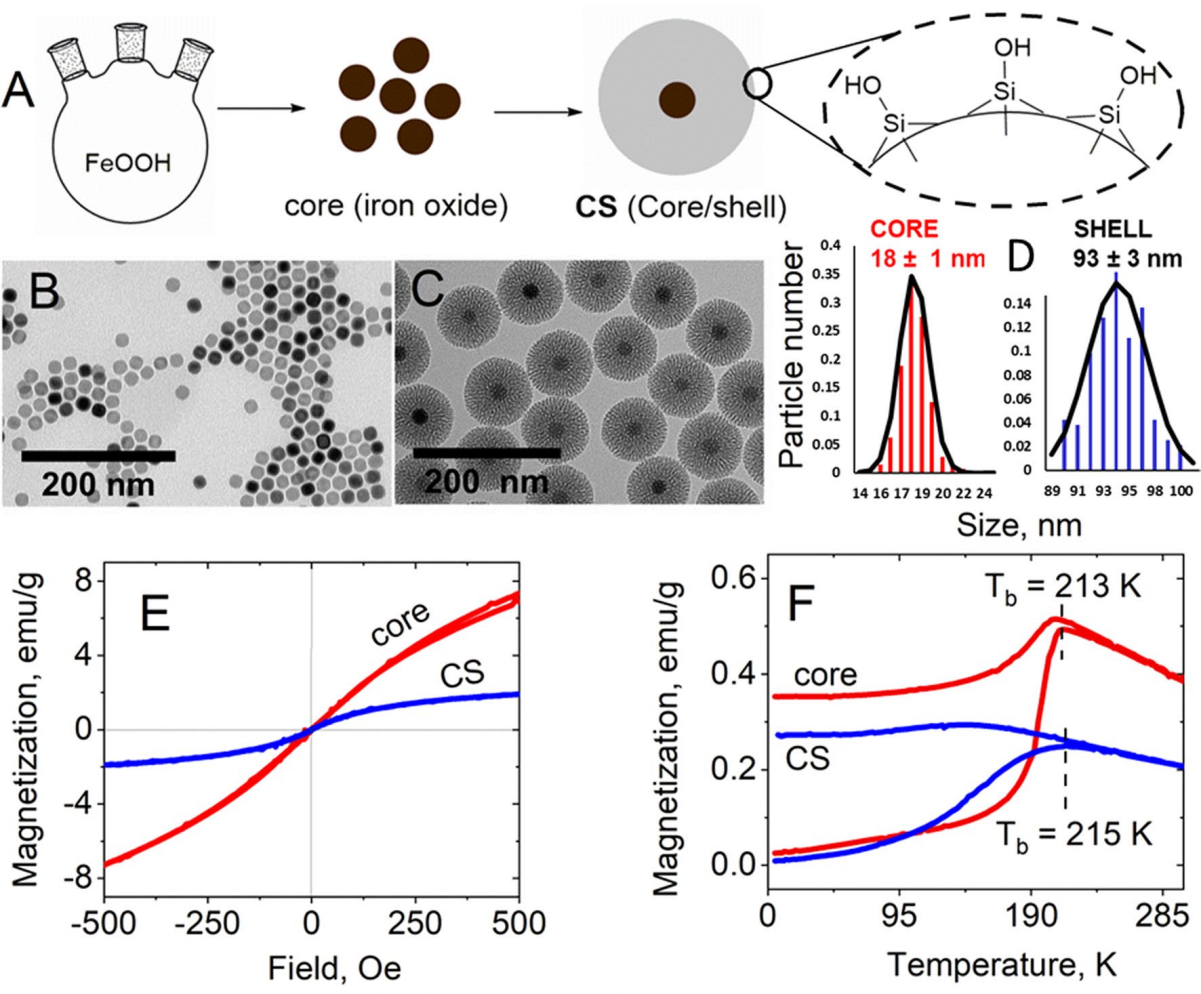
**A** scheme of synthesis of particles with iron oxide core and mesoporous silica shell. Silica surface is represented in detail on the right. **B** and **C** TEM images of iron oxide (core) and core with silica shell (core/shell) nanoparticles. **D** size distribution of the core and core/shell nanoparticles is analyzed from data of TEM, using 10 diameter classes [33]. A mean diameter of 93 ± 3 nm is obtained for silica shell with a Particle Dispersion Index PDI = 0.14. Its core size has a mean diameter of 18 ± 1 nm with PDI = 0.11. Assuming silica density of 2.2 g/cm^3^ and iron density of 4.9 g/cm^3^, the volume of each nanoparticle (4.21** × **10^–22^ m^3^) is composed of the iron volume of 3.05** × **10^–24^ m^3^ and the silica volume of 4.18** × **10^–22^ m^3^. For each composite nanoparticle, the mean iron mass is 1.50** × **10^–20^ kg and the mean silica mass is 9.20** × **10^–19^ kg. **E** magnetization curves of core and core/shell nanoparticles recorded at room temperature versus the applied field. **F** magnetization measurements of core and core/shell particles as a function of temperature with an applied field of 20 Oe

As shown in Fig. 1A, the magnetic core and core/shell particles were fabricated by the decomposition of iron oxide hydrate and sol–gel reaction with tetraethyl orthosilicate, respectively [29].

These nanoparticles were inspected using TEM imaging (Fig. 1B–C). TEM images showed that core/shell particles are significantly larger than core particles, both being monodispersed. A single magnetic core was observed, at the center of a porous silica shell. Figure 1D shows the size distribution of the MNPs and core/shell particles, determined from TEM images at low magnification images. It evidences a narrow size distribution for both particle types, the mean size of MNPs and core/shell being determined about 18 nm and 93 nm, respectively. Magnetic core displayed magnetite phase as published by our group [29] that make it is compatible for biological application due to the widely occurrence of magnetite crystals in the living organism.

To understand the magnetic properties of these particles, the hysteresis loop and FC-ZFC curves of the two particles (few mg of samples) were measured at room temperature using a SQUID magnetometer (Quantum Design MPMS-XL). Figure 1E shows hysteresis loops of the samples in the field range of – 500–500 Oe. Both samples have a small coercivity field (< 1 Oe, in the zoom-in profiles). Therefore, the particles exhibited superparamagnetic property at room temperature. At 500 Oe, the magnetization of MNPs and core/shell was 7.3 emu/g and 1.9 emu/g, respectively. The shell is diamagnetic, with a very much weak magnetization compared to superparamagnetism of the iron core. The susceptibilities $\left( {\chi_{v} = \frac{M}{H}} \right)$ of core and core/shell particles were 0.183 × 10^–3^ m^3^/kg and 0.048 × 10^–3^ m^3^/kg, respectively.

Thermal magnetization behaviors in the zero field (ZFC)—field cooled (FC) were performed from 4 to 300 K, under an applied magnetic field of 20 Oe Fig. 1F. The ZFC curves were obtained by cooling the samples in zero magnetic field from 300 to 4 K. Then, at 4 K a magnetic field of 20 Oe was applied, and the magnetization named ZFC curves was recorded with increase in the temperature to 300 K. Thus, the FC curves were recorded using the same protocol except that the samples were cooled from 300 K under the same field of 20 Oe. The ZFC–FC curves show the typical superparamagnetic profiles below an irreversibility temperature (*T*_i_) of around 250 K and a well-defined maximum in the ZFC curves [34] at the so-called blocking temperature *T*_b_ of 213 and 215 for core and CS (core/shell) nanoparticles, respectively. However, the maximum of ZFC curve for core/shell NPs is very broad suggesting non-negligible dipole–dipole magnetic interactions [34] among iron oxide cores nanoparticles (mean diameter of 18 nm) even when they are encapsulated in mesoporous silica shell (mean diameter of 93 nm). Moreover, as shown in Fig. 1F, for the iron -oxide cores nanoparticles, the FC curves present a usual behavior such as a strong decrease of the magnetization with decreasing the temperature. This result may indicate the existence of strong interparticle antiferromagnetic or ferrimagnetic dipole interaction among iron oxide nanoparticles leading a decrease of the magnetization with decreasing the temperature as reported in a recent study [34].

The low *T*_b_ of the core/shell particles (− 58 ℃) meets very well the requirements of bio-applications at positive temperatures, *i.e*., much higher than *T*_i_ = 250 K, by ensuring that the CS particles will be in a superparamagnetic state and will therefore not aggregate, while retaining magnetization. The combination of Fe_2_O_3_ core and nanostructured Silica not only leads to interesting properties of nanocomposite system, but is also a good candidate for applications in biomedicine because both are biocompatible in biological systems due to their low toxicity and biodegradability.

Based on the works of Wagner & Fisher [35] and Leong et al. [36] (Additional file 1), we provide theoretical arguments to explain that composite nanoparticles with superparamagnetic core behave as simple silica nanoparticles as soon as magnetic field is not applied. According to the formula of Calderon et al. [37] developed in the (Eq. S14), the addition of a thickness of 2 × 37.5 nm of mesoporous silica shell between two magnetite cores divided by 712-fold the maximal attractive magnetic force in the presence of a constant magnetic field, avoiding or reducing aggregation behavior.

### Structure elucidation of silanized *trans*-resveratrol derivatives

The covalent bond process of the silane-derivative linker and *trans*-resveratrol molecules was inspected by observing the appearance of the C=O group at 1700 cm^−1^ and the disappearance of –N=C=O group at 2270 cm^−1^ in the FT-IR transmittance spectra (Additional file 1: Fig. S1). It is to be noted that, as compound **1** was always the dominant product even at the early stage of the reaction, we used an excess of *trans*-resveratrol compared to (triethoxysilyl)propyl isocyanate to obtain compound **3.** The structures of three main products (Additional file 1: Fig. S2) were elucidated by NMR (Additional file 1: Fig. S5–Fig. S26) and their molecular weight were determined by mass spectroscopy (Additional file 1: Fig. S27–S30).

In the ^1^H-NMR spectra of **1**, all signals of three OH groups disappeared at 9–10 ppm. However, only one signal of the hydrogen atom of -OH group at 9.65 ppm was observed in the ^1^H-NMR spectrum of **2,** and two –OH signal at 9.20 was observed in compound **3**. These data allow us to confirm that –OH functional positions of *trans*-resveratrol frameworks in **1, 2,** and** 3** were modified with three, two, and one substituted groups, respectively. Also, the signals of –NH group emerged at 7.7–7.8 ppm in the ^1^H-NMR spectra, and signals of C=O bond at 154 ppm in the ^13^C-NMR spectra are the evidence for the existence of the carbamate group of **1, 2,** and** 3**. The signals of linear hydrocarbons in the structure of 3-(triethoxysilyl)propyl isocyanate were found on the ^1^H-NMR spectra at 1–4 ppm and ^13^C-NMR spectra at 7–60 ppm with the proper ratio for 3, 2, and 1 substituted groups.

Precisely, the two-dimension NMR spectra of compounds **2** and **3** were tested to pinpoint the –OH conjugated position. The signal of –OH group appearing at 9.65 ppm on the proton spectrum of compound **3** proved that the two hydroxyl groups remained on the A ring of *trans*-resveratrol. Moreover, HMBC spectra showed the coupling of C-4′ with H-3′ and H-5′ and the drop of the chemical shift of C-4′ from 157.4 ppm to 151.1 ppm revealed the substitution of –OH at C-4′ position of compound **3.** The structure of compound **2** was elucidated by the key couplings between protons and carbons on the HMBC spectrum: (1) –OH (9.65 ppm) with C-4, and C-6 proved the presence free of hydroxyl group on the A ring; (2) H-4 (6.41 ppm), H-2 and H-6 with C-3 and C-5 show that one hydroxyl group of A ring is substituted and makes the imbalance of A ring; therefore, C-3 and C-5 have the different chemical shift, 154.3 ppm, and 158.2 ppm, respectively; (3) H-3′ and H-5′ with C-4′ on the B ring prove the –OH at C-4′ is modified with the chemical shift decrease of C-4′ from 157.4 ppm in the *trans*-resveratrol structure to 150.7 ppm of compound **2**.

In conclusion, NMR characterization showed that compound (**1**) corresponds to 3′,4,5′-tri((3-(triethoxysilyl)propyl)carbamate)-*trans*-stilbene, compound (**2**) is 5′-hydroxy-4-((3-triethoxysilyl) propyl)carbamate)-3′-bis((3-(triethoxysilyl)propyl)carbamate)-*trans*-stilbene, and finally, compound (**3**) is 3′,5′-dihydroxy-4-((3-(triethoxysilyl)propyl)carbamate)-*trans*-stilbene.

### Functionalization of nanoparticles with silane-*trans*-resveratrol derivatives

**Fig. 2:**
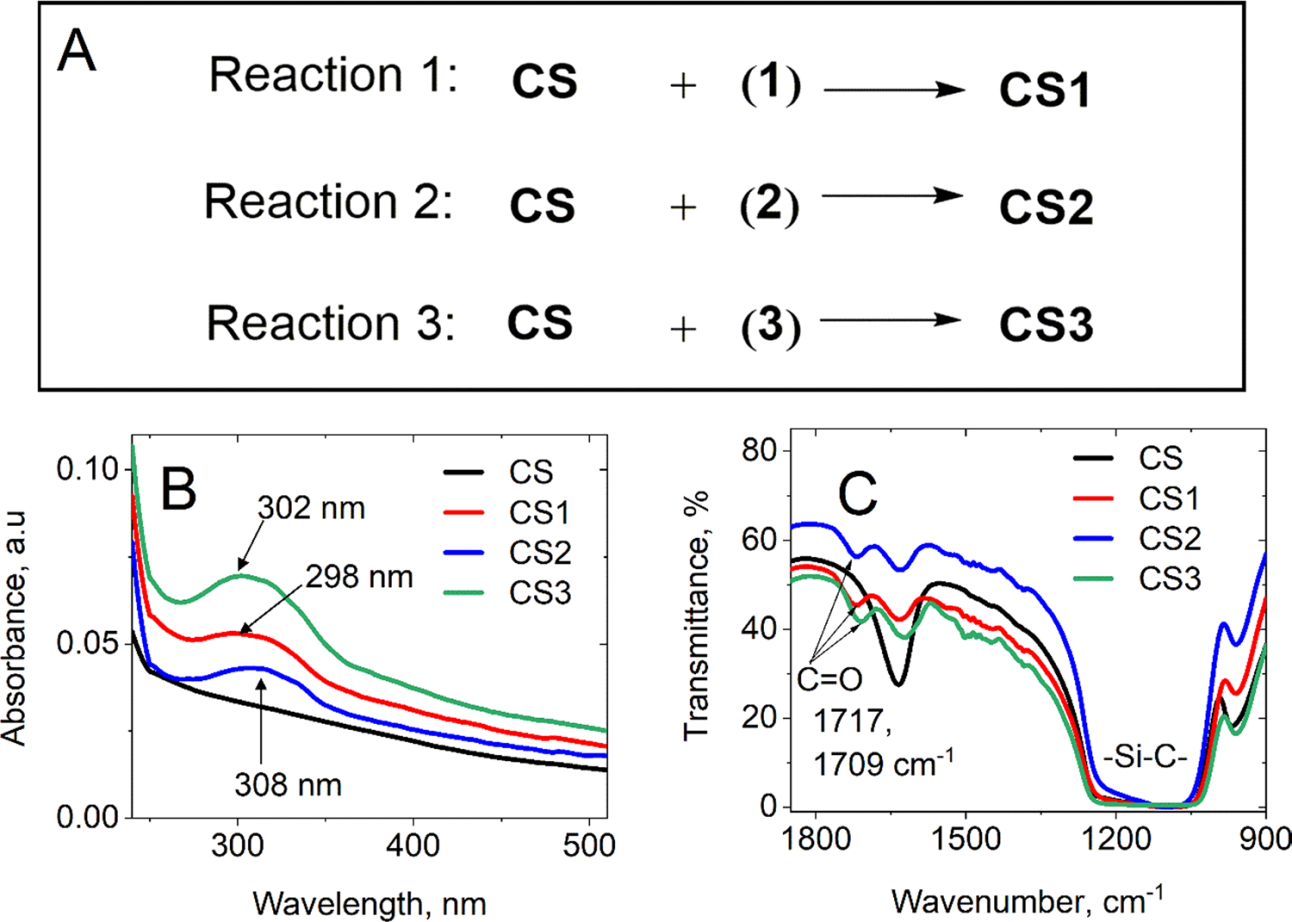
**A** Scheme of synthesis of **CS** nanoparticles grafted with *trans*-resveratrol. **B** UV–Vis spectra of the 3 types of grafted nanoparticles from 240 to 510 nm.** C** The infrared spectra of core/shell grafted with **1**, **2**, **3** molecules corresponding to **CS1**, **CS2**, and **CS3**, respectively. Both UV–Vis and IR spectra of native **CS** (black curves) were plotted to observe the changes in absorbance and transmittance properties of the nanoparticles

To evaluate the success of *trans*-resveratrol grafting on nanoparticles, UV–Vis spectra were recorded (Fig. 2B). Three distinct spectra with maximum wavelengths at 298 nm, 308 nm, and 302 nm were observed for **CS1**, **CS2,** and **CS3**, respectively, which corresponded to the absorbance band of *trans*-resveratrol [38, 39]. More detailed analyses of UV-cross section extinction spectra are provided in Fig. 5 and will be discussed later.

In parallel, we compared the FT-IR transmittance spectra of **CS1**, **CS2**, **CS3,** and the pristine **CS** in Fig. 2C. In the four spectra*,* similar bands were observed at 900–1200 cm^−1^ and 1634 cm^−1^_,_ corresponding to the Si–C bond overlap with the Si–O band and the deformation of the vibration of adsorbed water molecules on the silica shell [40], respectively. Additional peaks at 1717 cm^−1^ (**CS1** and **CS2**) and 1709 cm^−1^ (**CS3**) corresponded to C=O bonds of in frameworks of **1**, **2**, **3**. These findings were consistent with a successful covalent coating of core/shell nanoparticles surfaces with *trans*-resveratrol. The slight differences between the wavenumber of the maximum absorption of the C=O band in the IR spectra CS**1**, **CS2**, and **CS3** and between the maximum wavelengths on the UV–Vis spectra could reflect conformation changes of silane-resveratrol derivatives when they were coated with a silica shell and/or the position of silane that was grafted. We observed that absorption maxima of CS1, CS2, and CS3 were between 280 and 340 nm. We observe that they are centered near the 304 nm corresponding to the main optical electronic HOMO–LUMO transition from Π → Π* of *trans*-resveratrol in water, assessed by theoretical DFT calculation [41].

### Impact of pH of the aqueous TRIS buffer on nanoparticle aggregation

**Fig. 3:**
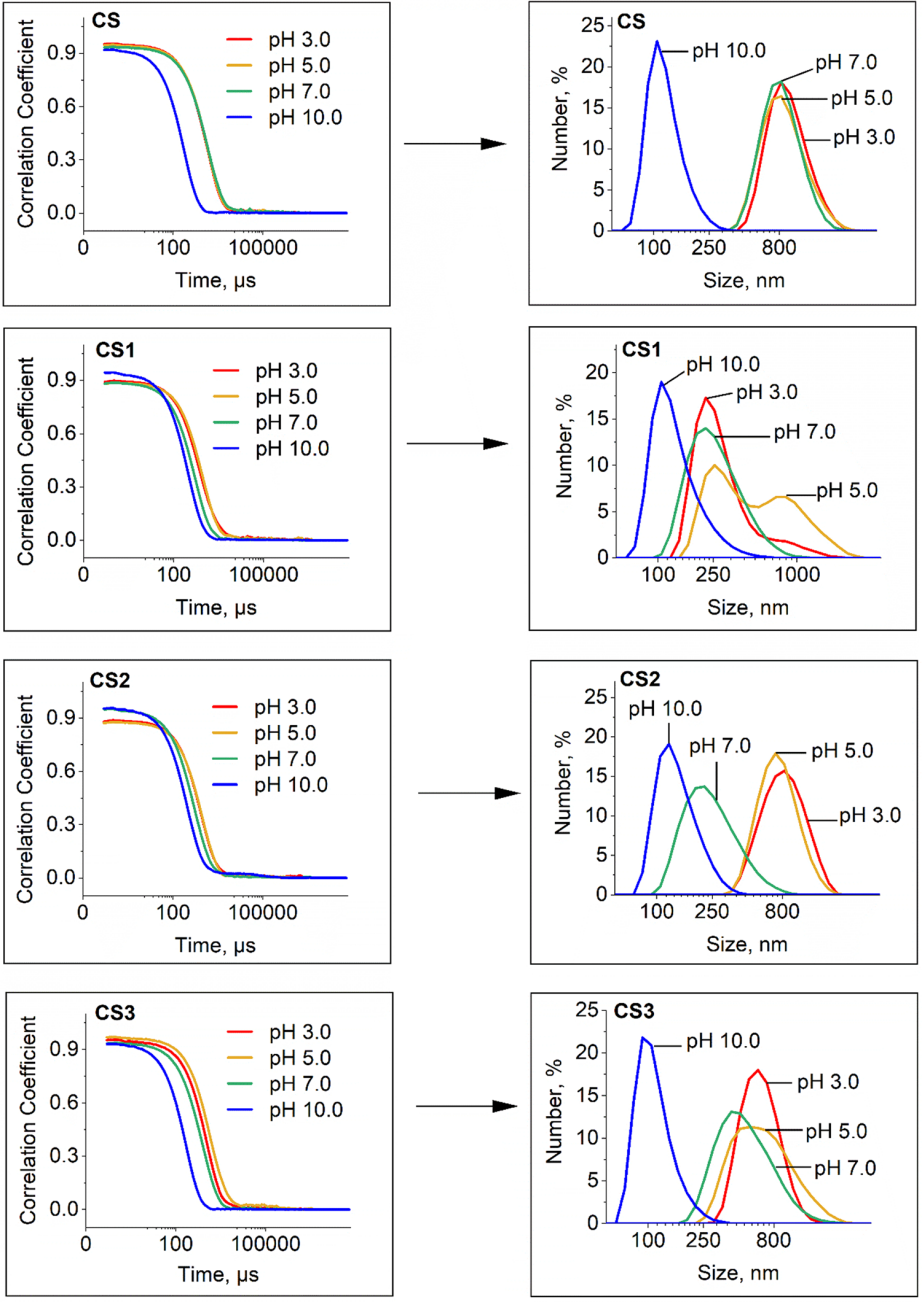
Size distribution of uncoated and coated nanoparticles in TRIS buffer 50 mM at different pH values, obtained by dynamic light scattering

DLS measurements were carried out to examine the colloidal stability of nanoparticles within the 3.0–10.0 pH range generally used for in *vitro* protein interaction studies**.** The correlation coefficient increases with nanoparticle size or upon particle aggregation, and which is associated with a slower diffusion speed of particles. For CS, the nearly single exponential decay of the autocorrelation function obtained by DLS suggests a narrow distribution of **CS** sizes in the suspension. Figure 3 evidenced that **CS, CS1, CS2** and **CS3** are monodisperse at pH 10.0. Resveratrol grafting onto **CS** has a negligible effect on the hydrodynamic radius (105, 120 and 100 nm for **CS1, CS2,** and **CS3,** respectively) at this alkaline pH value. At pH 10.0, nanoparticles dispersed in absolute ethanol had the same size distribution as in TRIS buffer, which evidenced their monodispersed colloidal behavior (see Additional file 1: Fig. S3). Since **CS1** has 3 silane groups grafted on silica shell, **CS1** may potentially react with one or two neighboring nanoparticles during the functionalization step, resulting in nanoparticle covalent dimers and trimers (clusters) of around 200 nm or 300 nm diameter, respectively, whereas the non-reacting **CS1** remains as a single nanoparticle of 95–100 nm diameter. Similarly, **CS2** has 2 silane groups and may form covalent dimers. The DLS analysis of **CS1** and **CS2** suspended in alkaline TRIS buffer (pH 10.0) revealed that dimers and trimers may exist only in very negligible proportions. **CS1**, **CS2,** and **CS3** populations can thus be considered as a population of single nanoparticles after their functionalization.

From neutral to acidic conditions, the *d*_*H*_ distribution of **CS** shifted drastically and widened up to 800 nm, evidencing an aggregation process during acidification. The highest *d*_*H*_ value was reached for the most acidic condition (Additional file 1: Fig. S4). Indeed, the dispersed state vanished as soon as pH decreased below 7.0. This aggregation was fully reversible when alkalinized with NaOH (data not shown). The three functionalization types resulted in three distinct aggregation behaviors. The mean *d*_*H*_ of aggregates depended on pH value, but differently, without conforming to one general pattern. An intermediate average diameter of aggregates was observed at pH 7.0 for **CS2**, and **CS3**.

**Fig. 4:**
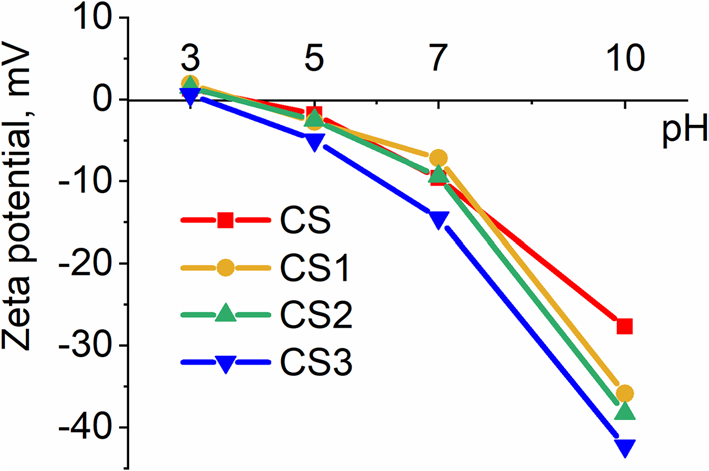
pH dependence of zeta potential (expressed in mV) at 25 °C of **CS**, **CS1**, **CS2** and **CS3** suspended in TRIS buffer (50 mM) at concentration of 50 ppm (~ 50 µg/mL), corresponding to a volume fraction of 2.25 × 10^–5^. Each point was the mean of 5 replicates

As shown in Fig. 4, the mesoporous silica core/shell nanoparticles synthesized in this work exhibited a weak positive zeta potential at pH 3.0, monotonously decreasing with increasing pH. In alkaline conditions, monodispersed **CS** exhibited a zeta-potential value of − 27.7 ± 6.4 mV. Functionalization by resveratrol-silane derivatives significantly increased the negative value of the zeta potential of **CS1**, **CS2,** and **CS3**. Using zeta-potential results of Fig. 4, surface charge density was estimated with equation S13, corresponding to an analytic solution of the Poisson–Boltzmann equation for charged sphere (Additional file 1: Fig. S34). At pH 3.0, surface charge density remained negligible for nanoparticles of the four types. Negative charge density increased almost exponentially with increasing pH between 3.0 and 10.0 (× 22, × 20, × 24, and × 77 fold increase for CS, CS1, CS2, and CS3, respectively). Since both zeta potential and surface charge density decreased less for CS than for functionalized nanoparticles (CS2, CS3), we can hypothesize that free hydroxyl of resveratrol could contribute to additional negative charge in alkaline conditions. However, this hypothesis does not support the high zeta-potential value of CS1 at pH10, as there is no hydroxyl group for ionization. At present, we ignore which silanol group type (isolated Q^3^, vicinal Q^1^, interacting or germinal Q^2^) is preferentially grafted to silanized resveratrol, which resveratrol hydroxyl(s) remain(s) free for CS2 and CS3. Additional investigations would be necessary to fully explain the observed pH-control of the surface charge density.

The most negative value was obtained for **CS3**, which contained the resveratrol-silane derivative with two free hydroxyls on one of its two phenolic groups and was also deprotonated at pH 10.0. In neutral and acidic conditions, the low zeta-potential values corresponded to those of nanoparticle aggregates of an average *d*_H_ up to 825 nm diameter.

**Fig. 5:**
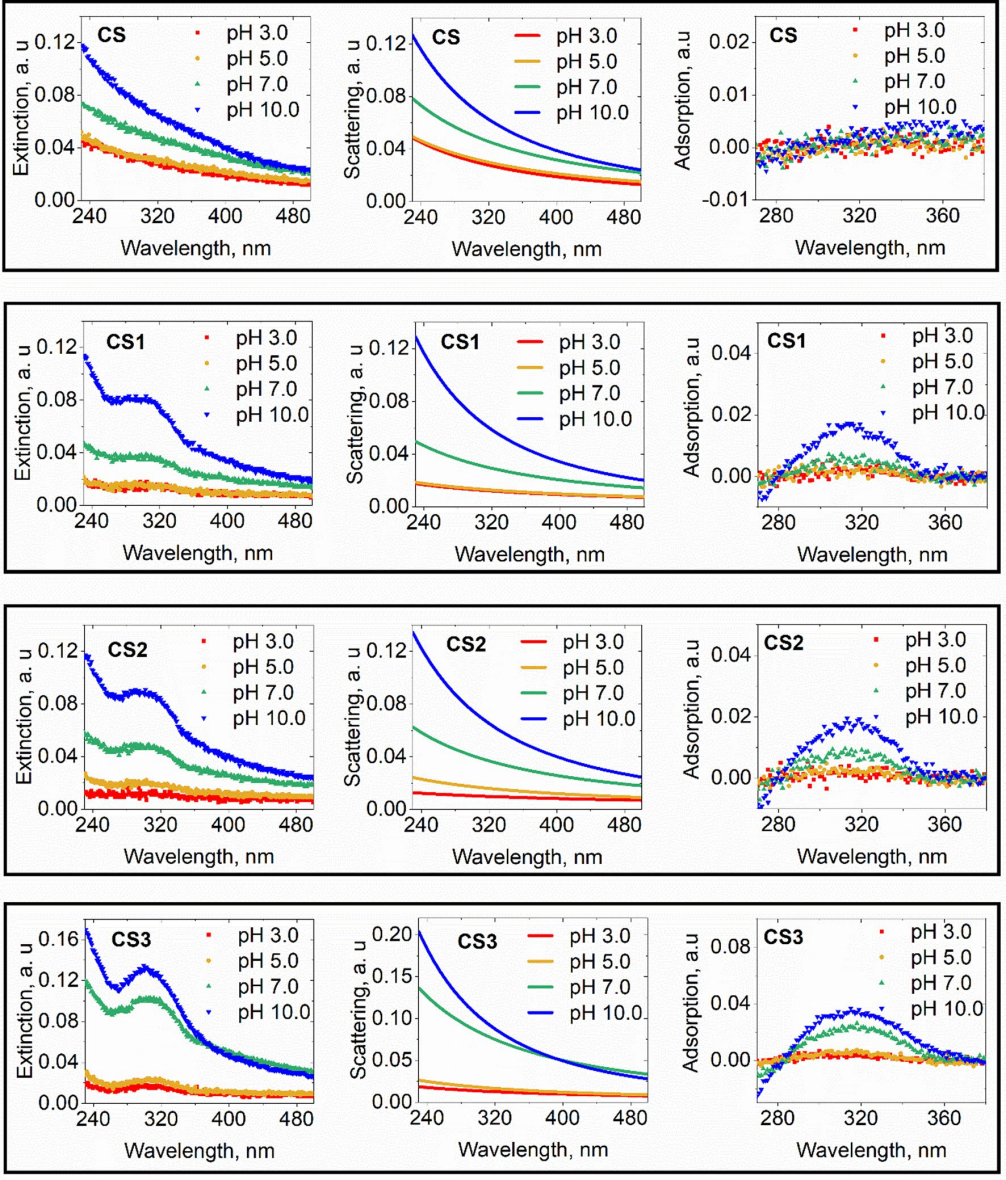
pH dependence of extinction cross section spectra of **CS, CS1, CS2**, and **CS3** recorded using an EnSpire microplate reader (left column), with the subtraction of TRIS buffer extinction cross section spectrum. In the central column, scattering cross section spectra were fitted to *A*_*s*_(*λ*) = *W* *λ*^−n^. In the right column, adsorption cross section spectra were deduced by subtraction of extinction to scattering cross section. Scattering fits were overestimated below 280 nm, so that negative values of small intensities occurred, showing that scattering and adsorption cross section could not be accurately assessed in the [230–280 nm] wavelength domain

Extinction cross section (*A*(*λ*)) spectra of **CS**, **CS1**, **CS2**, and **CS3** have been recorded both in clear bottom microplates using the EnSpire plate reader in absorbance mode (Fig. 5) and in quartz cuvettes with a UV-1800 spectrophotometer (not shown), giving equivalent results. Scattering cross section (*A*_s_(*λ*)) spectra of **CS** conformed to an exponential decrease of the wavelength for all the TRIS buffer pH values tested, fitting very well to the Rayleigh–Gans–Debye model [42] by Eq. (1):

1 $$A_{s} \left( \lambda \right) = W \cdot \lambda^{ - n}$$

Constants *W* and *n* were deduced from the linear fitting of the logarithm of extinction as a function of the logarithm of wavelength (Additional file 1: Table S1). Extinction cross section intensity *A*(*λ*) increased with pH value for a wavelength between 240 and 500 nm (Fig. 5). Scattering fits were underestimated below 280 nm (Fig. 5), with small negative values, showing that scattering and adsorption cross section could not be accurately assessed in the [230–280 nm] wavelength domain with the EnSpire reader plate. We also noticed that compared to **CS**, silane-resveratrol-grafted nanoparticles displayed an additional absorbance maximum centered at 315–320 nm. The adsorption value increased with pH, which is also the case of free *trans-*resveratrol between pH 5.0 and 10.0 [43]. The adsorption maximum was different for the three grafted types due to the different substitution and grafting.

## Discussion

**Fig. 6:**
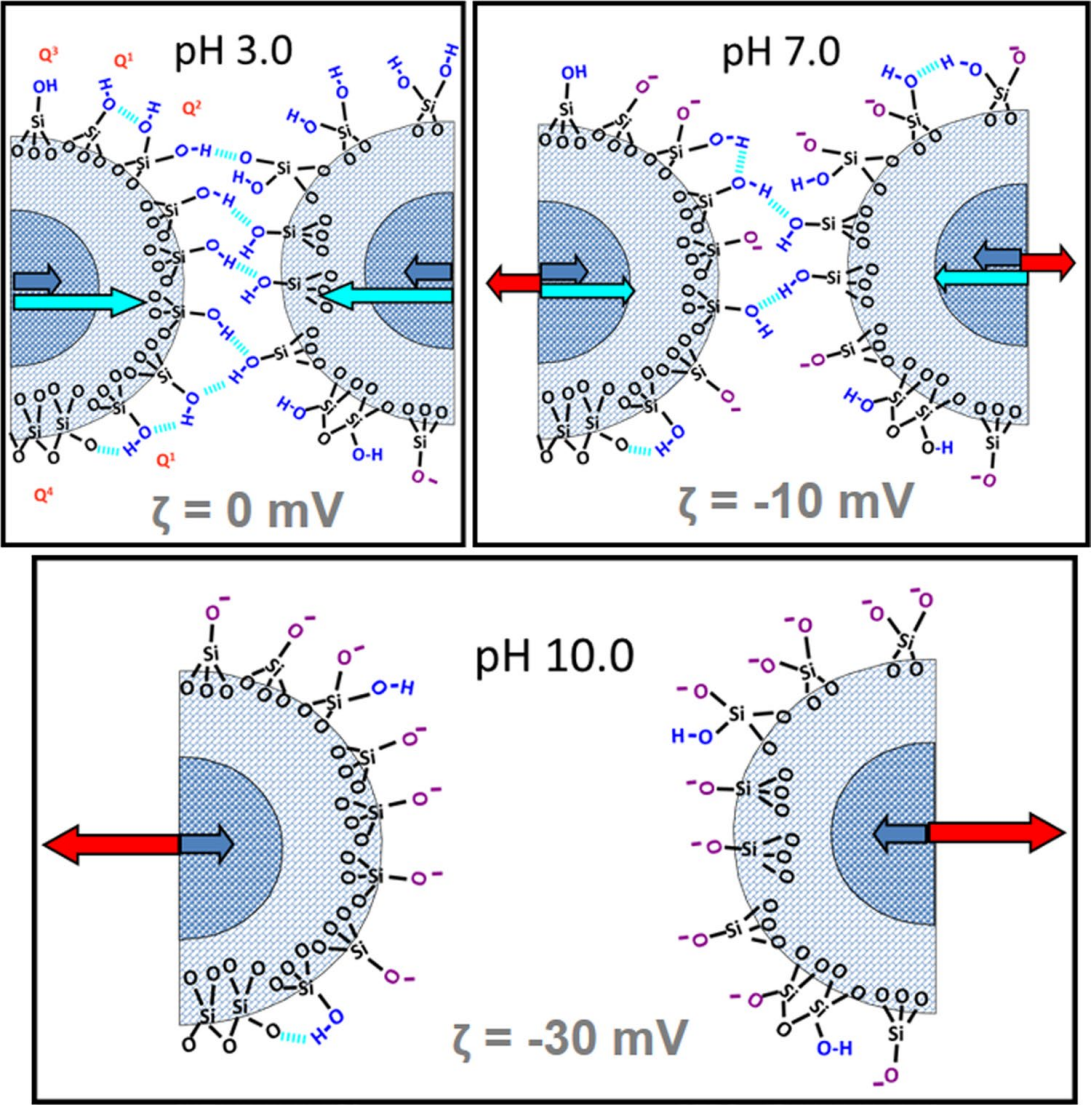
pH dependence of the aggregation behavior of nanoparticles in TRIS 50 mM buffer (p*K*a 8.3). At pH 3.0 (zeta potential ***ζ*** ~ 0), all silanols are protonated and van der Waals attractive forces between nanoparticles (blue arrow) are reinforced by interparticle hydrogen bonds (green arrow) formed between silanols. Solely a few isolated silanols (Q^3^), which have the lowest p*K*a, are deprotonated. At pH 7.0 (***ζ*** = − 10 mV), isolated (Q^3^), vicinal (Q^1^) and germinal (Q^2^) silanols tend to be deprotonated so that a well-formed counter-ion layer exerts a repulsive force (red arrow) on nanoparticles. Interparticle hydrogen bonds also decrease at pH 7.0. At pH 10.0 (***ζ*** = − 30 mV), all silanols are deprotonated and the counter-ion layer is fully developed, exerting higher repulsive forces, and preventing aggregation. The magnetic attraction force is negligible due to superparamagnetism property at room temperature

Mesoporous silica exhibits four types of silanol groups, which can be separately studied by both NMR [44, 45] and vibrational infrared spectroscopy [46]. They exhibit contrasted acidity and chemical reactivity [47–49]. Single silanol groups, designated as isolated silanols (Q^3^), with three covalent Si–O links with the bulk silica, are located at a far distance from neighboring H-donors or H-acceptors, which prevents hydrogen bonding. Q^3^ are the most acidic silanols (p*K*_a_ around 4.8) and therefore become the first deprotonated sites at pH 5.0. They are followed by hydrogen-bonded vicinal (Q^1^) and germinal (Q^2^) silanols, which have an intermediary acidity (8.5 < p*K*_a_ < 9.3) and are progressively deprotonated between pH 7.0 and pH 10.0. Finally, inner silanols (Q^4^) are the least acidic ones (p*K*_a_ > 11.0). They thus remained always protonated in the pH range of our experiments (3.0 and 10.0) [49] (Fig. 6).

Consequently, as observed on Fig. 4 describing the pH dependence of the zeta potential, the surface charge density of **CS** remained very reduced at pH 3.0, and therefore, the electrical double-layer repulsion of counter-ions had a very weak strength. Then, attractive forces between nanoparticles such as van der Waals dispersion forces became sufficient for spontaneous nanoparticle aggregation [50]. Depending on the three functionalization types studied, phenolic groups of two neighboring nanoparticles could also be submitted to additional interaction forces, in particular *π*–*π* stacking forces. They may explain differences in aggregation processes (reaction-limited and diffusion-limited cluster aggregation) and in morphology (compactness, shape, gyration radius), resulting in contrasted *d*_*H*_ distributions between **CS1**, **CS2,** and **CS3**. In the case of **CS3**, the presence of the resorcinol group at their surface may also create a specific affinity site for the silica surface of the neighboring nanoparticles [51]. Indeed, catechol, a position isomer of resorcinol, is adsorbed more easily than water onto silica surface due to the specificities of its hydrogen bonding [52].

The distribution of the more acidic silanol groups on the mesoporous silica surface may be possibly heterogeneous, at the origin of the puzzle of more negative areas and more positive areas as pH increased. Electrostatic forces increased with the deprotonation of the surface silanols and may participate in repulsive forces between nanoparticles. Finally, when the majority of Q^2^ and Q^3^ silanols became deprotonated, the surface charge density became high and the long-range electrostatic repulsion of counter-ions became strong enough to stabilize the water suspensions in a monodispersed state, even at high ionic strength. This pH-dependent charge density of **CS** was observed in the case of many metal oxides: α-quartz and α-cristobalite [53], α-alumina [54], smectite [55], illite [56], magnetite Fe_2_O_3_ [57], maghemite Fe_2_O_3_ [58], titanium oxide (Ti0_2_) [57], zinc oxide (ZnO) [57].

Interestingly, the zeta potential of coated nanoparticles became more negative than the **CS** net charge with the increase of the pH value. The surface charge of metal oxides originates from four major mechanisms [57]: (1) the isomorphous substitution of structural Si^4+^ by cations of lower valence (Al^3+^, Fe^3+^, Zn^3+^…) creates structural charges which are pH-independent; (2) under-coordinated Si^4+^ of silica layer edges hydrate into silanols in an attempt to complete their coordination sphere. Usually, siloxanes of surface layers are potentially partially oxidized in silanol when they experienced a rich O_2_ medium. Protonations/deprotonations of interfacial silanols generate charges depending on the aqueous pH buffer; (3) some charges also originate from additional lattice imperfections and impurities; (4) finally, interfacial complexation of protons or other ions also occurs within the inner-sphere or the outer-sphere complexes.

To assess colloidal stability of our nanoparticles CS, CS1, CS2, and CS3, we added in Additional file describing step by step the DLVO equations that we apply, including the magnetic aggregative energy, and its extended XDLVO form (Additional file 1: Fig. S31 with parameters of Additional file 1: Table S2). The pH dependence of colloidal behavior of composite magnetite core with silica shell is badly predicted by DLVO model. When Lewis acid–base interaction energy is added to take into account polar interactions of silanols at silica interface, the prediction of the XDLVO conforms with DLS results (Additional file 1: Fig. S32 and S33). The energy barrier, which prevents aggregation, was calculated from DLVO and XDLVO simulations (Additional file 1: Fig. S33) with parameters of Additional file 1: Table S2. The energy barrier in acidic and neutral conditions decreases under the value of 15 kBT units, which is usually considered as the aggregation limit. CS1, CS2, and CS3 form stable suspensions in alkaline conditions above pH 9.0, and CS becomes stable above pH 10.0.

In a future work, using small amounts of nanoparticles, vibrational infrared spectroscopy could be used to assess silanol proportions. Indeed, changes in their proportion should induced changes in absorption bands at 3747 cm^−1^ (isolated silanol), at 3660 and 3620 cm^−1^ (vicinal silanol), at 3740 cm^−1^ (germinal silanol), and at 1110, 1050, 1085 cm^−1^ (siloxane) [59]. Using many nanoparticles, solid-state NMR of ^29^Si could reveal if the number of silanols and their Q^2^–Q^3^–Q^4^ proportions are modified after the three functionalizations (**CS1**, **CS2**, **CS3**), resulting in an average p*K*_a_ shift. Finally, potentiometric acidity titration may also highlight surface charge density of functionalized nanoparticles.

## Conclusion

This work reports the preparation of novel magnetic nanoparticles grafted with *trans*-resveratrol derivatives and their aggregation behavior in an aqueous environment. Thanks to their superparamagnetic property at room temperature, MNPs coupled with *trans*-resveratrol are expected to be good candidates for protein–ligand fishing and immunoassay design. Three *trans*-resveratrol derivatives were synthesized, purified and their structures were identified before their grafting on the surface of MNPs. This was an essential step for further understanding the interactions of these MNPs with protein. Since *cis*-resveratrol exhibits a maximum absorbance at 280–285 nm [38], we deduced from the UV–Vis spectra that *trans*-resveratrol was not isomerized into *cis*-resveratrol during the preparation process. The colloidal behavior/aggregation of these nanoparticles in aqueous solution is mainly governed by pH. They are monodispersed at pH 10.0 and tend to aggregate at neutral and acidic pH, due to the decrease of the surface charge density (assessed by zeta-potential measurement), resulting in lower electrostatic repulsion forces. The aggregation process results in the decrease of the extinction cross section, mainly due to the decrease of the light scattering cross section. A negligible difference in aggregation behavior was observed between uncoated and coated nanoparticles. The understanding of the aggregation behavior of nanoparticles in aqueous solution at different pH is important for their uses in quantitative applications.

Monodisperse MNPs are expected for protein–ligand fishing and immunoassay design. Consequently, we may hypothesis that the proposed functionalizations have to be improved for these applications. Fluorescence binding analysis of these MNPs with CpLIP2 lipase was carried out in acidic, neutral and basic pH by Nguyen [60], and revealed that fluorescence resonance energy transfer (FRET) occurs between some tryptophans of the proteins (as FRET donors) and silica-linked resveratrols (as FRET acceptors). Surprisingly, nanoparticle aggregation does not affect both protein binding constant and binding kinetics. Nguyen [60] demonstrated that even in acidic conditions, aggregates are small enough and loosely compact to affect protein-resveratrol interactions. This result opens up application opportunities even in small aggregate conditions.

## Supplementary Information

**Additional file 1**: **Table S1**. The parameters of the Rayleigh-Gans-Debye model.** Table S2**. Parameters.** Fig. S1**. Transmission spectra of silanized trans-resveratrol.** Fig. S2**. Structure of silanized trans-resveratrol.** Fig. S3**. Size distribution of nanoparticles.** Fig. S4**. Distribution of zeta potential of nanoparticles at different pH.** Figs. S5** to **S26**. NMR spectra of compound 1, 2, and 3.** Fig. S27**. Mass spectroscopy of compound 1, 2, and 3.** Figs. S28** to **S30**. MALDI-TOF spectra of compounds 1 to 3.** Fig. S31**. Interaction energy of two spherical nanoparticles in aqueous TRIS buffer calculated using DLVO and XDLVO models.** Fig. S32**. Potential energy of interaction of two spherical nanoparticles.** Fig. S33**. Energy barrier of colloidal suspension of nanoparticles.** Fig. S34**. Surface charge density of silica nanoparticles.

## Data Availability

All of the material is owned by the authors. The datasets of this study are available from the corresponding author upon reasonable request.
