# Supplementary material for: Aggregation of magnetic nanoparticles functionalized with trans-resveratrol in aqueous solution
Source: Discov Nano. 2023 Apr 19;18(1):64. doi: 10.1186/s11671-023-03805-9 (PMC10409977; doi:10.1186/s11671-023-03805-9)
Supplement: Supplementary file 1 — Additional file 1: Table S1. The parameters of the Rayleigh-Gans-Debye model. Table S2. Parameters. Fig. S1. Transmission spectra of silanized trans-resveratrol. Fig. S2. Structure of silanized trans-resveratrol. Fig. S3. Size distribution of nanoparticles. Fig. S4. Distribution of zeta potential of nanoparticles at different pH. Figs. S5 to S26. NMR spectra of compound 1, 2, and 3. Fig. S27. Mass spectroscopy of compound 1, 2, and 3. Figs. S28 to S30. MALDI-TOF spectra of compounds 1 to 3. Fig. S31. Interaction energy of two spherical nanoparticles in aqueous TRIS buffer calculated using DLVO and XDLVO models. Fig. S32. Potential energy of interaction of two spherical nanoparticles. Fig. S33. Energy barrier of colloidal suspension of nanoparticles. Fig. S34. Surface charge density of silica nanoparticles. [file 11671_2023_3805_MOESM1_ESM.docx]

**Aggregation of magnetic nanoparticles functionalized with *trans*-resveratrol in aqueous solution**

*Thi-Nga Nguyen^1,2,3^, Quang-Hung Tran^2,4^, Ferial Terki^2^, Clarence Charnay^5^, Xavier Dumail^5^, Corinne Reibel^5^, Guillaume Cazals^6^, Gilles Valette^6^, Christian Jay-Allemand^1^, Luc P. R. Bidel^7^*

*^1^UMR IATE, University of Montpellier, Institut Agro, INRAE, F-34060 Montpellier, France.*

*^2^PhyMedExp UMR CNRS 9214 – Inserm U1046, 34295 Montpellier Cedex 05, France.*

*^3^Institute of Natural Products Chemistry, Vietnam Academy of Science and Technology, 18 Hoang Quoc Viet, 100000 Hanoi, Vietnam.*

*^4^eV-Technologies, 2 Esplanade Anton Philips, Bâtiment 5, 14460 Colombelles, France.*

*^5^Institut Charles Gerhardt UMR 5253 CNRS-UM, Université de Montpellier, F-34095 Montpellier, France.*

*^6^IBMM UMR5247, CNRS, ENSCM, Université de Montpellier, Place Eugène Bataillon, 34095 Montpellier Cedex 5, France*

*^7^UMR AGAP, University of Montpellier, CIRAD, INRAE, Institut Agro, Montpellier, France.*

Corresponding: [luc.bidel@inrae.fr](mailto:luc.bidel@inrae.fr) and [ferial.terki@umontpellier.fr](mailto:ferial.terki@umontpellier.fr)

## **Additional file 1. Table S1**: The parameters of the Rayleigh-Gans-Debye model. **Table S2**: Parameters. **Fig. S1.** Transmission spectra of silanized *trans*-resveratrol. **Fig. S2**: Structure of silanized *trans*-resveratrol. **Fig. S3**: Size distribution of nanoparticles. **Fig. S4**: Distribution of zeta potential of nanoparticles at different pH. **Fig. S5 to S26**: NMR spectra of compound 1, 2, and 3. **Fig. S27**: Mass spectroscopy of compound 1, 2, and 3. **Fig. S28 to S30.** MALDI-TOF spectra of compounds 1 to 3. **Fig. S31**: Interaction energy of two spherical nanoparticles in aqueous TRIS buffer calculated using DLVO and XDLVO models. **Fig. S32**: Potential energy of interaction of two spherical nanoparticles. **Fig. S33**: Energy barrier of colloidal suspension of nanoparticles. **Fig. S34**: Surface charge density of silica nanoparticles.

##

## .

**Table S1**: The parameters of the Rayleigh-Gans-Debye model $A_{s}(\lambda)=W.\lambda^{-n}$deduced from the linear fitting of the logarithm of extinction as a function of the logarithm of the wavelength.

| pH | Compound | W | n |
| --- | --- | --- | --- |
| 3.0 | **CS1** | 8.68 | 1.14 |
|  | **CS2** | 0.86 | 0.77 |
|  | **CS3** | 10.65 | 1.17 |
| 5.0 | **CS1** | 9.65 | 1.15 |
|  | **CS2** | 23.70 | 1.26 |
|  | **CS3** | 60.79 | 1.42 |
| 7.0 | **CS1** | 298.61 | 1.60 |
|  | **CS2** | 378.79 | 1.60 |
|  | **CS3** | 2618.78 | 1.81 |
| 10.0 | **CS1** | 59006.52 | 2.39 |
|  | **CS2** | 19760.59 | 2.19 |
|  | **CS3** | 233292.08 | 2.57 |

| **Table S2**  **Parameters** | **Value** | **Units** |
| --- | --- | --- |
| Mean radius of the spherical colloid particle 1 (rp_1_) (usually rp_1_≤ rp_2_) | 4.65E-08 | m |
| Mean radius of the spherical colloid particle 2 (rp_2_) | 4.65E-08 | m |
| Rp=rp_2_/rp_1_ | 1 | - |
| Mean iron core radius | 9.00E-09 | m |
| Mean volume of the spherical particle CS | 4.212E-22 | m^3^ |
| Mean iron volume of the single nanoparticle CS | 3.054E-24 | m^3^ |
| Mean silica volume of the single nanoparticle CS | 4.181E-22 | m^3^ |
| Iron density | 4.9 | g / cm^3^ |
| Silica shell density | 2.2 | g / cm^3^ |
| Mean iron weight of the single nanoparticle CS | 1.496E-20 | kg |
| Mean silica weight of the single nanoparticle CS | 9.198E-19 | kg |
| Mean density of the single nanoparticle CS | 2219.58 | kg / m^3^ |
| Magnetic permeability of the vacuum (µ_0_) | 1.25664E-06 | H / m |
| Mass magnetization of a single iron core (1 A m^2^ /kg = 1 emu / g) | 4.489E-18 | A m^2^ / kg |
| Surface potential of the silica shell particle 1 (Ψ_p1_) | -0.0045 to 0.00128 | V |
| Surface potential of the silica shell particle 2 (Ψ_p2_) | -0.0045 to 0.00128 | V |
| Contact angle of material "1" (β_1_) | 26.4 | ⁰ |
| Contact angle of material "1" or "3" (β_3_) | 49 | ⁰ |
| Combined Hamaker constant (A_123_ or A_121_) | 2.23E-20 | J |
| Hydrophobic force constant (K_123_ or K_121_) | 7.97597E-21 | J |
| Characteristic wavelength (λ) | 0.0000001 | m |
| Born collision parameter (σ_Born_) | 5E-10 | m |
| Decay (Debye) length of the suspending liquid (λ_AB_) | 1 | nm |
| "Contact" distance (h_0_) | 0.157 | nm |
| Lewis acid-base free energy of interaction (Φ_ΑΒ_ (h=h_0_)) | -0.008085451 | J m^-2^ |
| Boltzmann constant (k_B_) | 1.4E-23 | J K^-1^ |
| Avogandro's number (N_A_) | 6.02E+23 | mol^-1^ |
| Fluid Absolute Temperature (T) | 303.15 | K |
| Dimensionless relative dielectric constant of liquid water (ε_r_) | 78.4 | - |
| Permittivity of free space (ε_0_) | 8.85E-12 | C2 J^-1^ m^-1^ |
| Elementary charge (e) | -1.602E-19 | C |
| Ionic strength (I_s_) | 0.0005 | mol L^-1^ |
| Inverse of the diffuse layer thickness (κ) | 72433302.47 | m^-1^ |

***Part 1. Silanization of trans-resveratrol***

**Figure S1**: Transmittance spectra of silanized *trans*-resveratrol as a function of the wavenumber of the reaction process at 7 hours and 3 days, recorded by attenuated total reflection of the infrared spectrometer.

Figure S2: Structure of silanized *trans*-resveratrol and their numbering of the carbon skeletons: 1, 2, 3 corresponding to the silane derivatives binds covalently to the three, two, and one hydrogen bonding sites of the *trans*-resveratrol molecule, respectively. Arrows show the key C-H correlations on the HMBC of compounds 2 and 3.

***Part 2. Dynamic light scattering measurement***


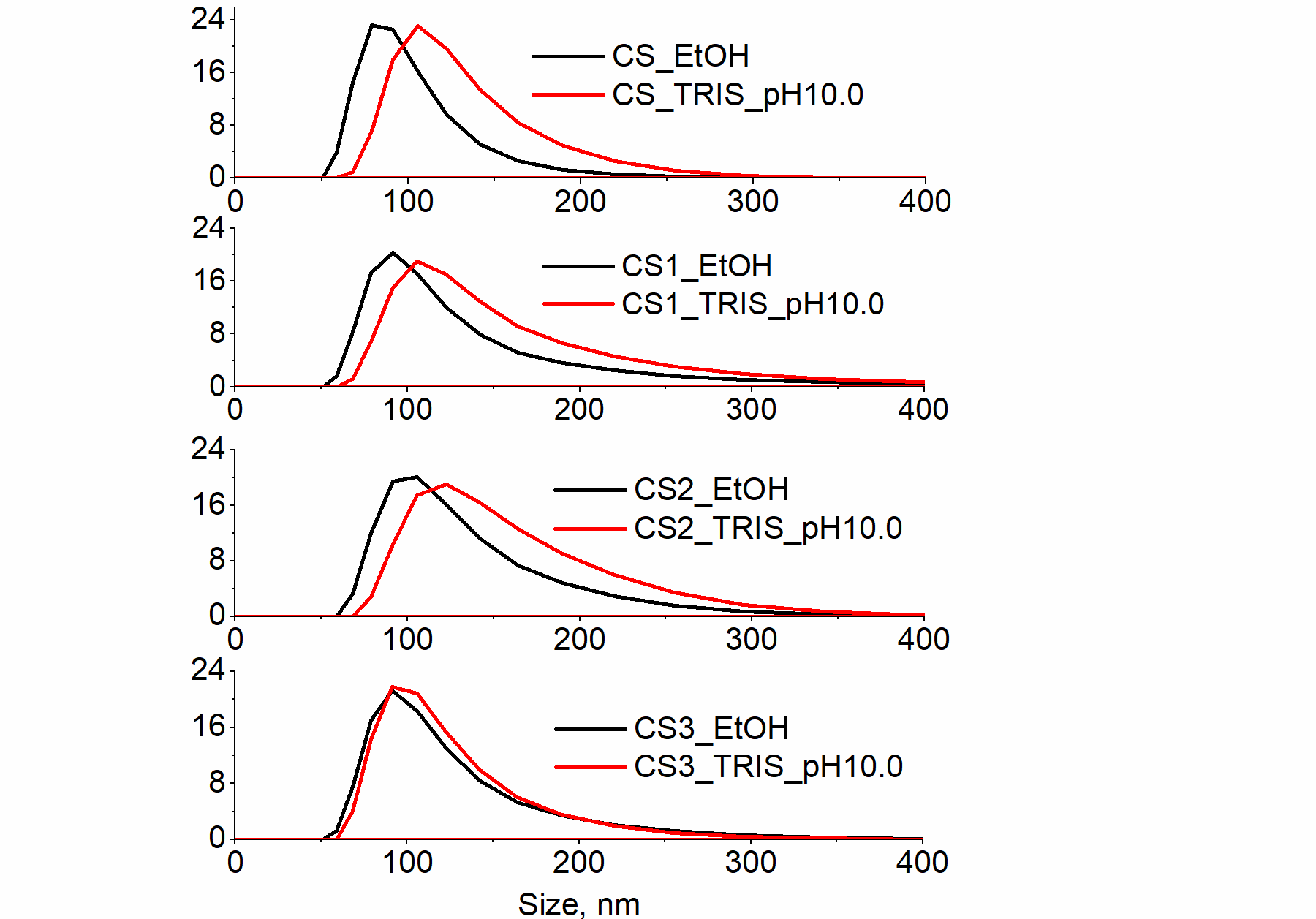


Figure S3: Size distribution of nanoparticles in absolute ethanol and in TRIS buffer at pH 10.0.

*Part 3. Zeta potential measurement*


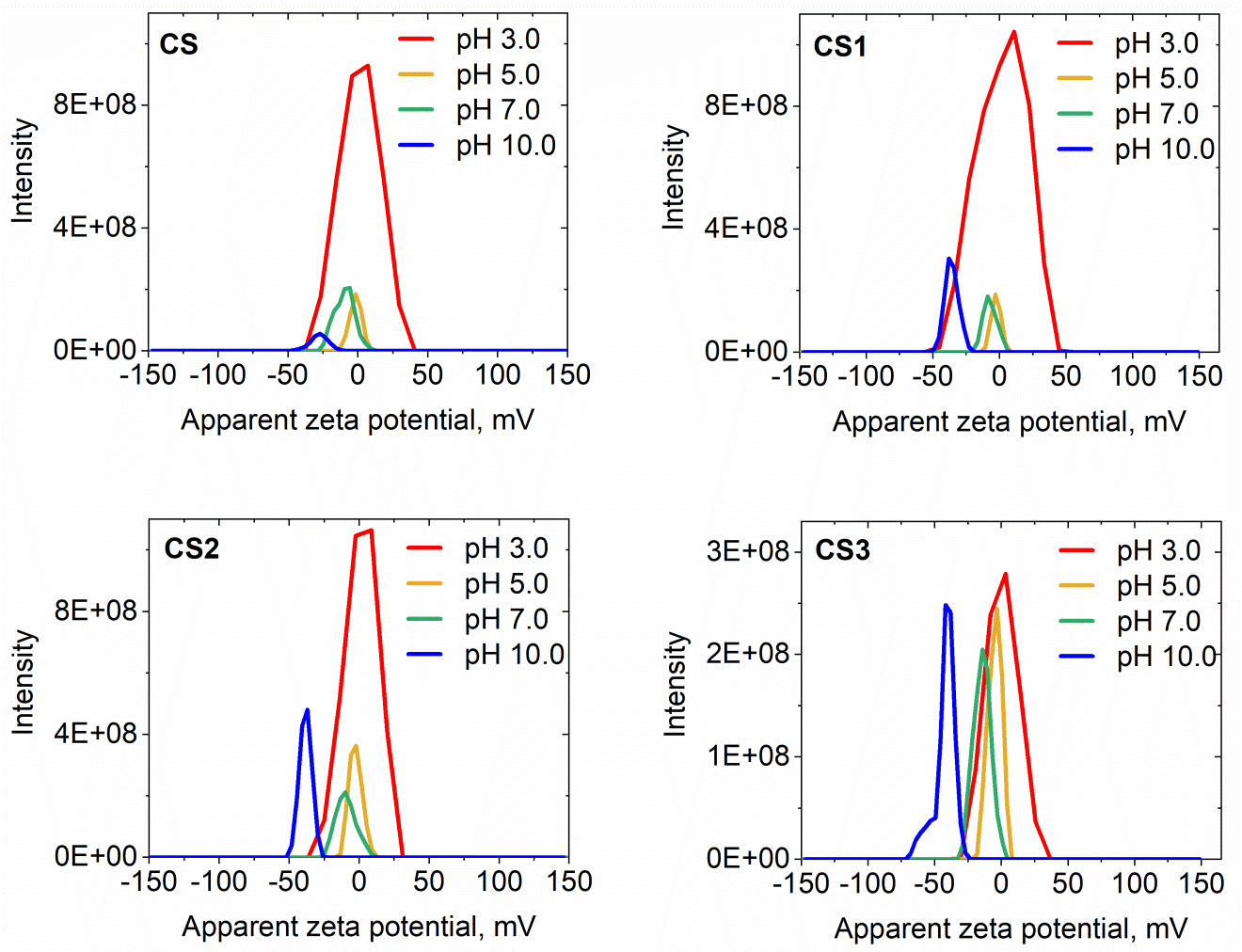


Figure S4: Distribution of zeta potential of nanoparticles at different pH.

***Part 4. Appendix NMR spectra of compound 1, 2, and 3***


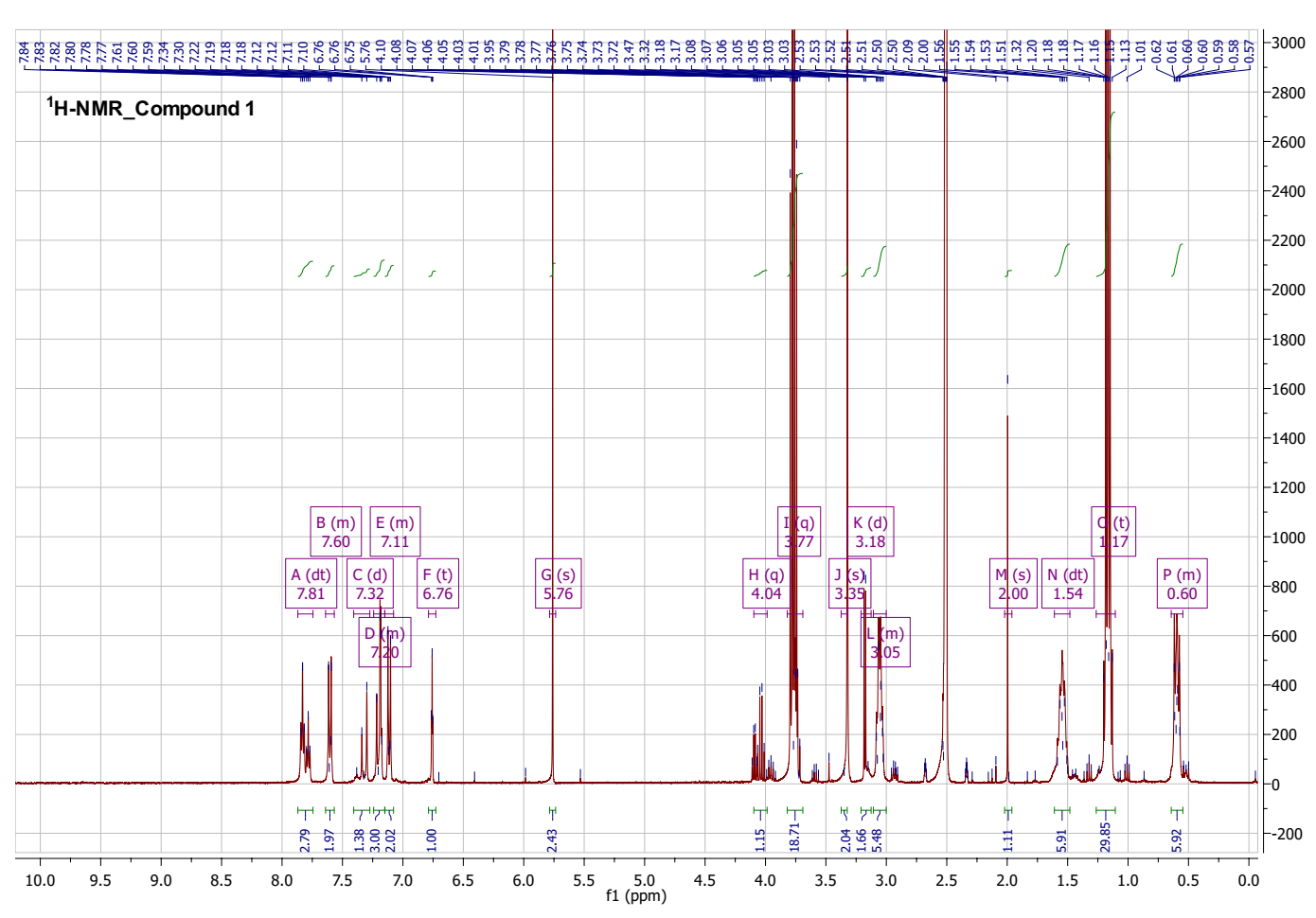


Figure S5: ^1^H-NMR spectrum of compound 1


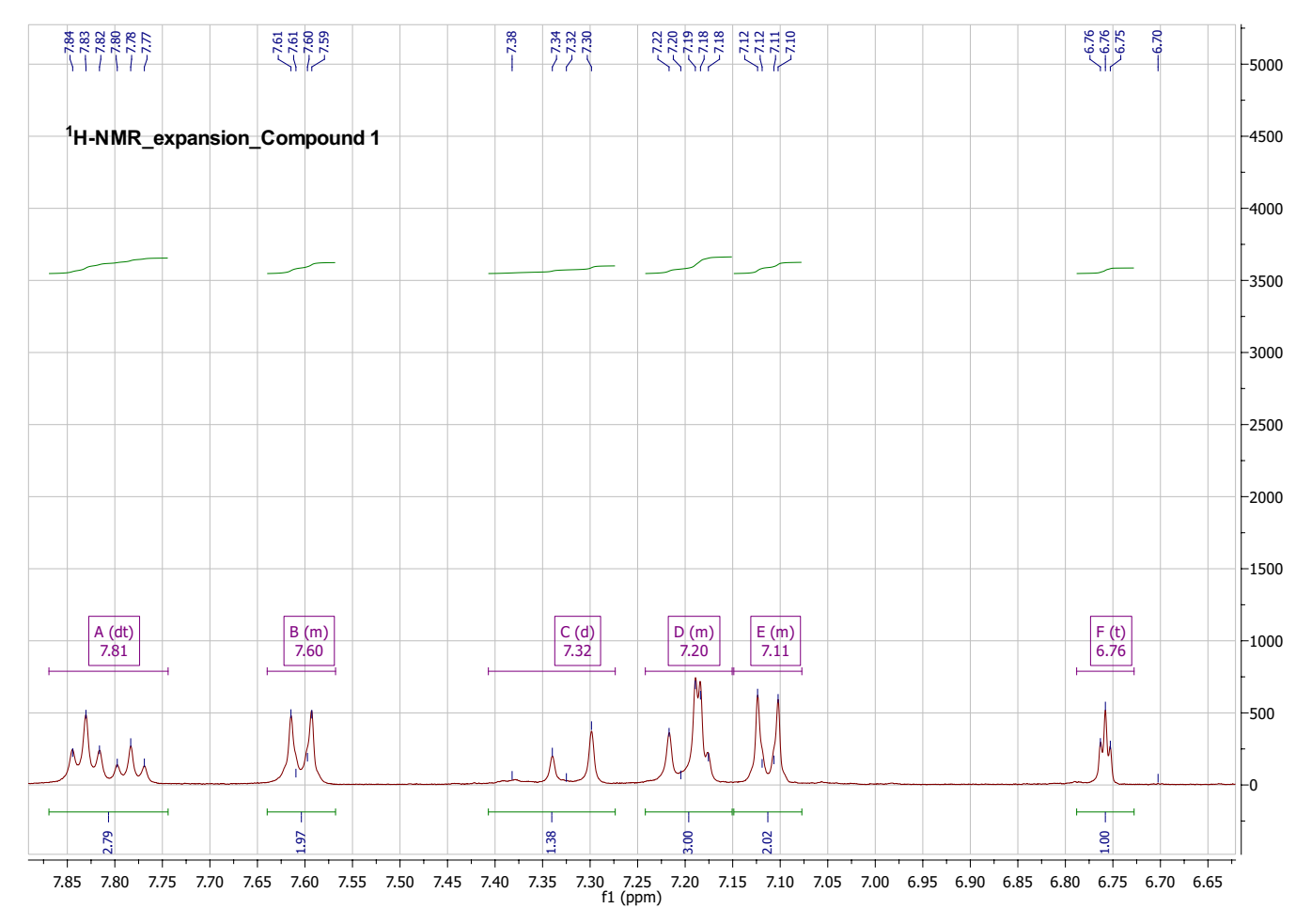


Figure S6: ^1^H-NMR spectrum of compound 1, expansion


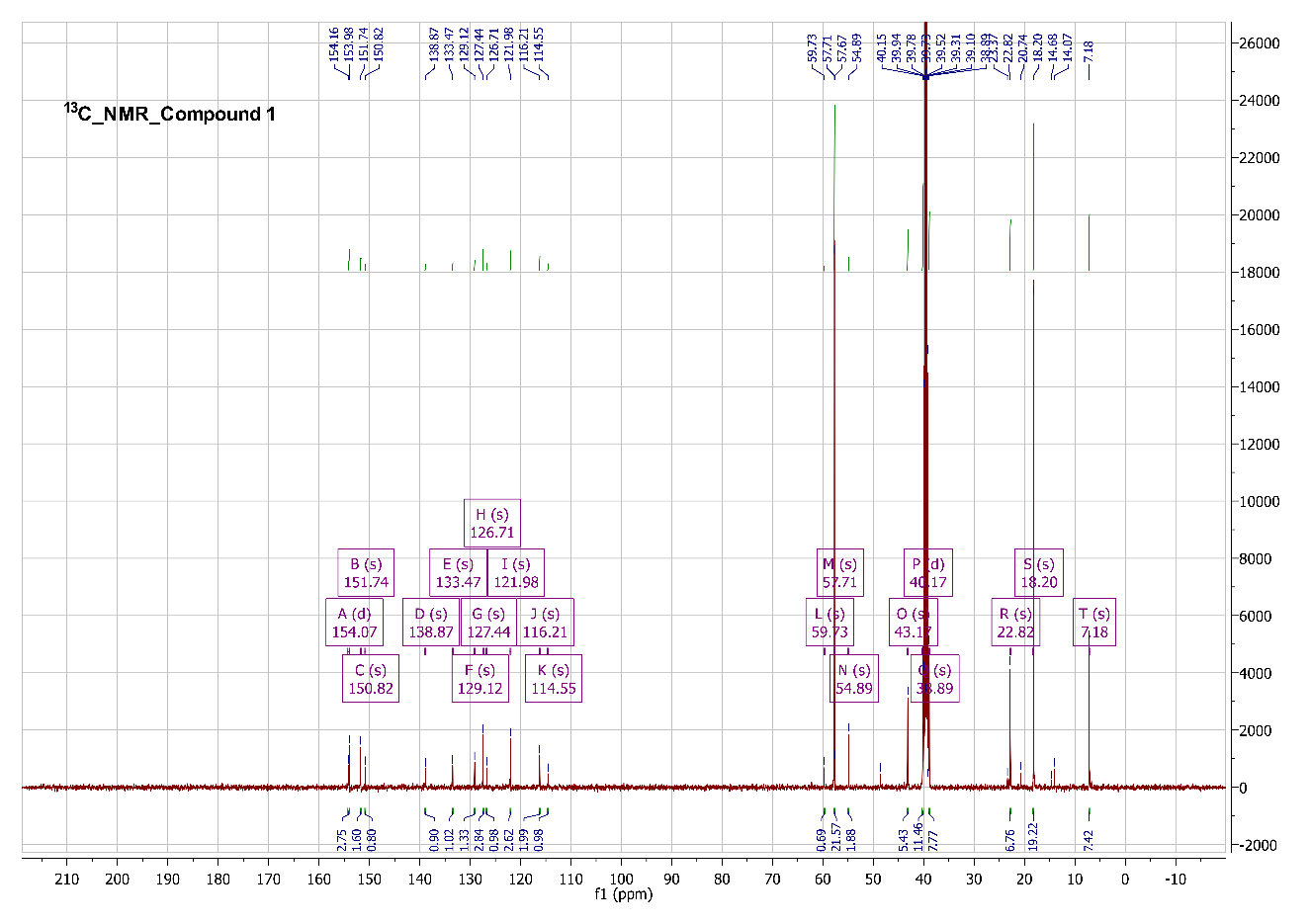


Figure S7: ^13^C-NMR spectrum of compound 1


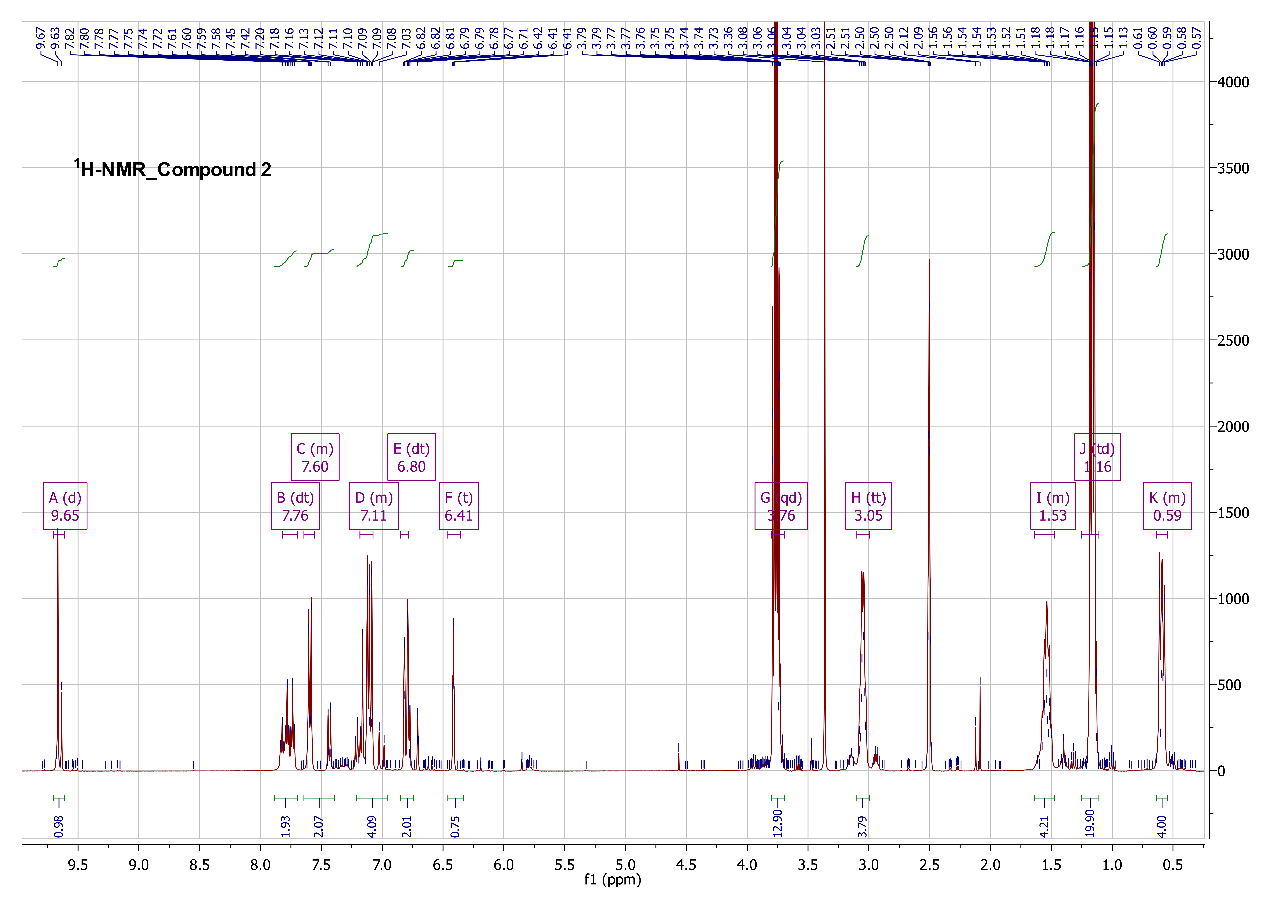


Figure S8: ^1^H-NMR spectrum of compound 2


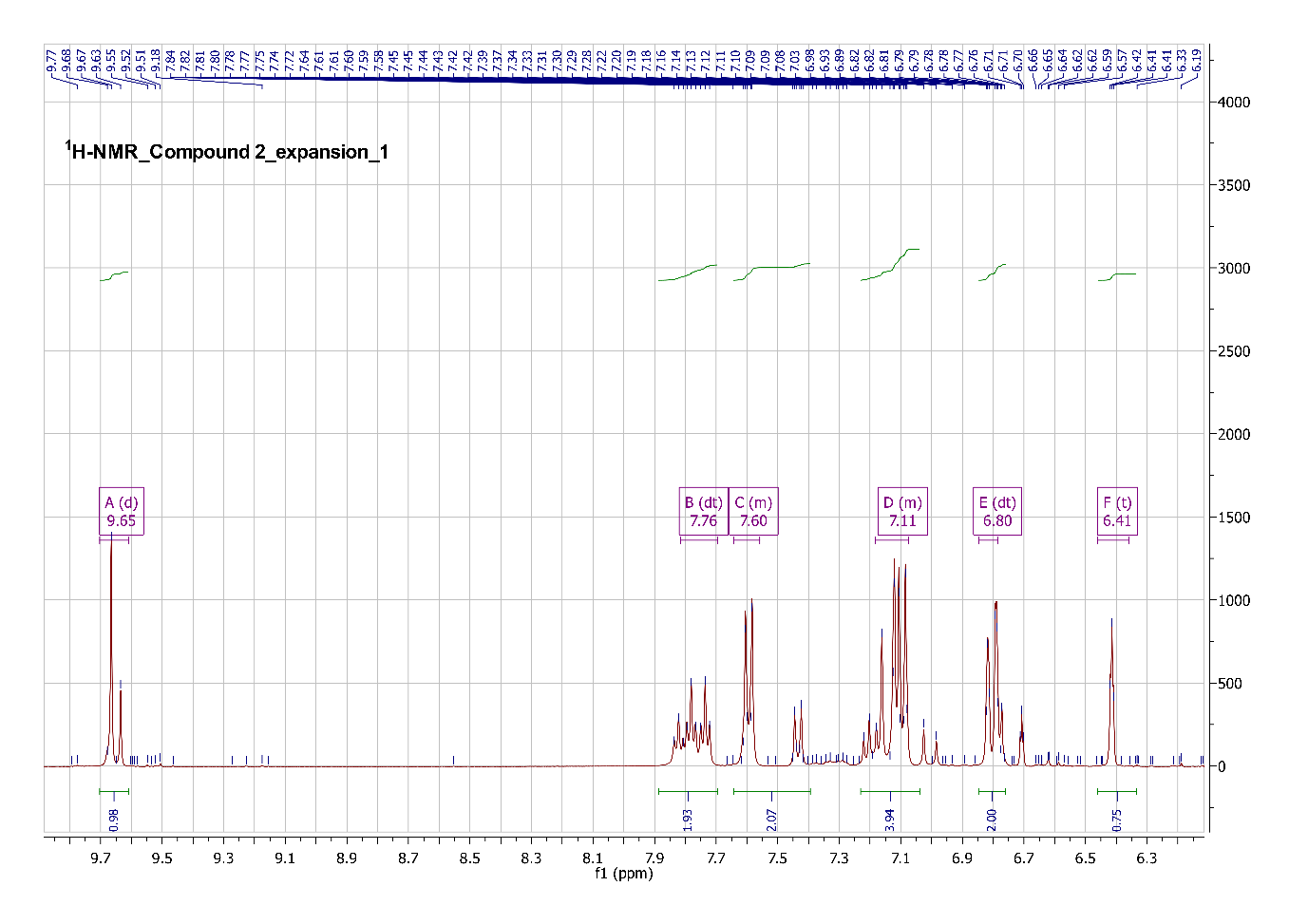


Figure S9: ^1^H-NMR spectrum of compound 2, expansion 1


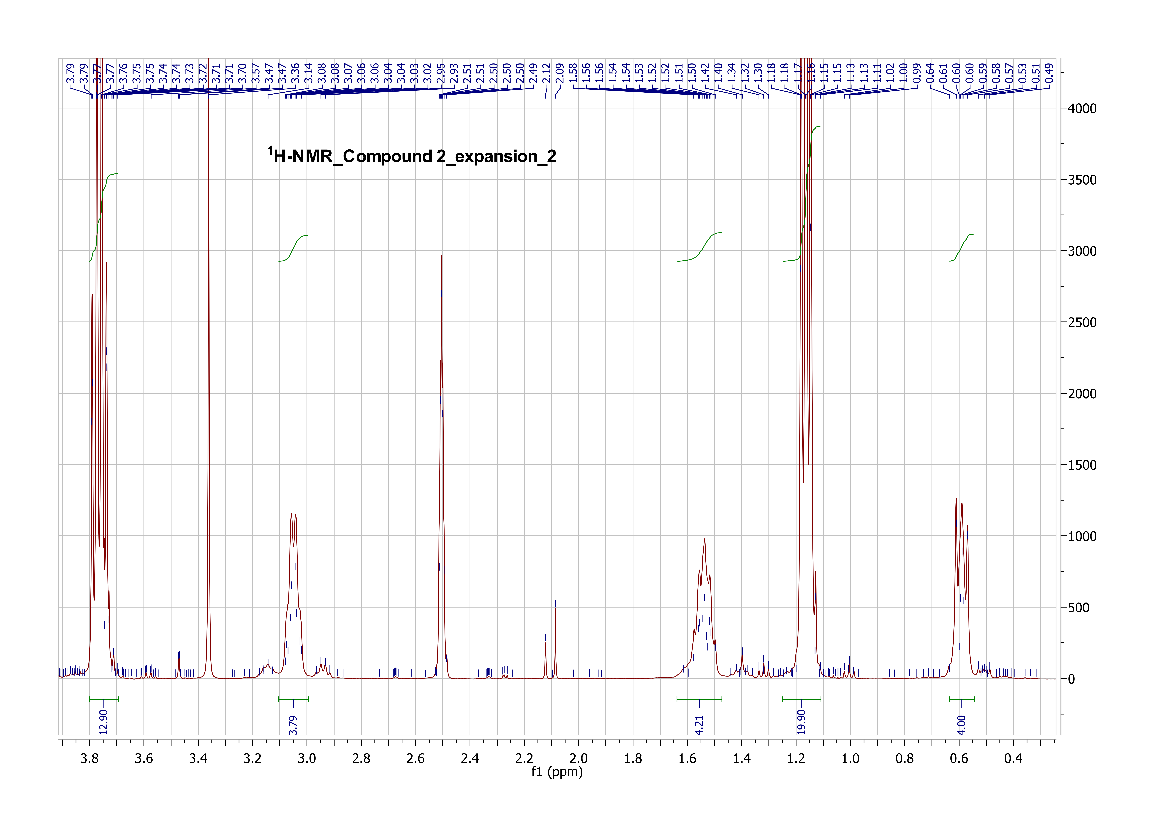


Figure S10: ^1^H-NMR spectrum of compound 2, expansion 2


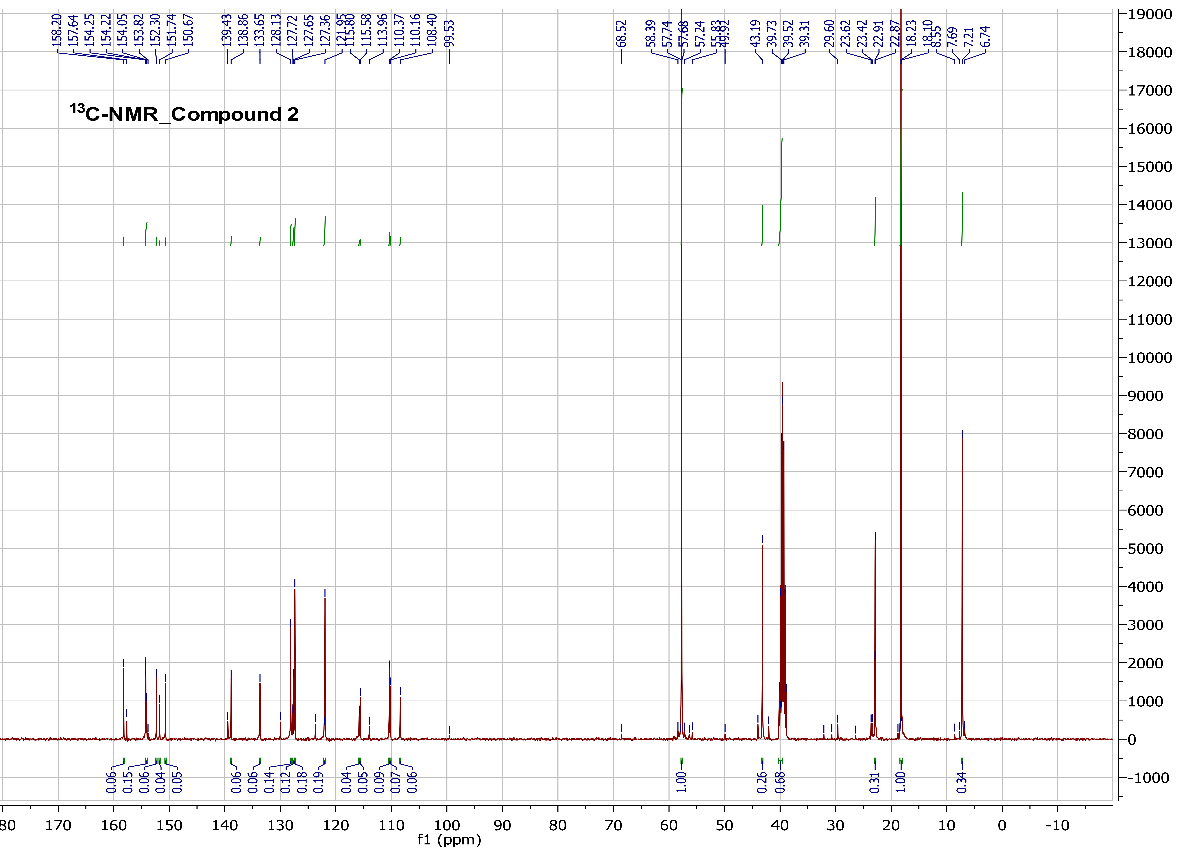


Figure S11: ^13^C-NMR spectrum of compound 2


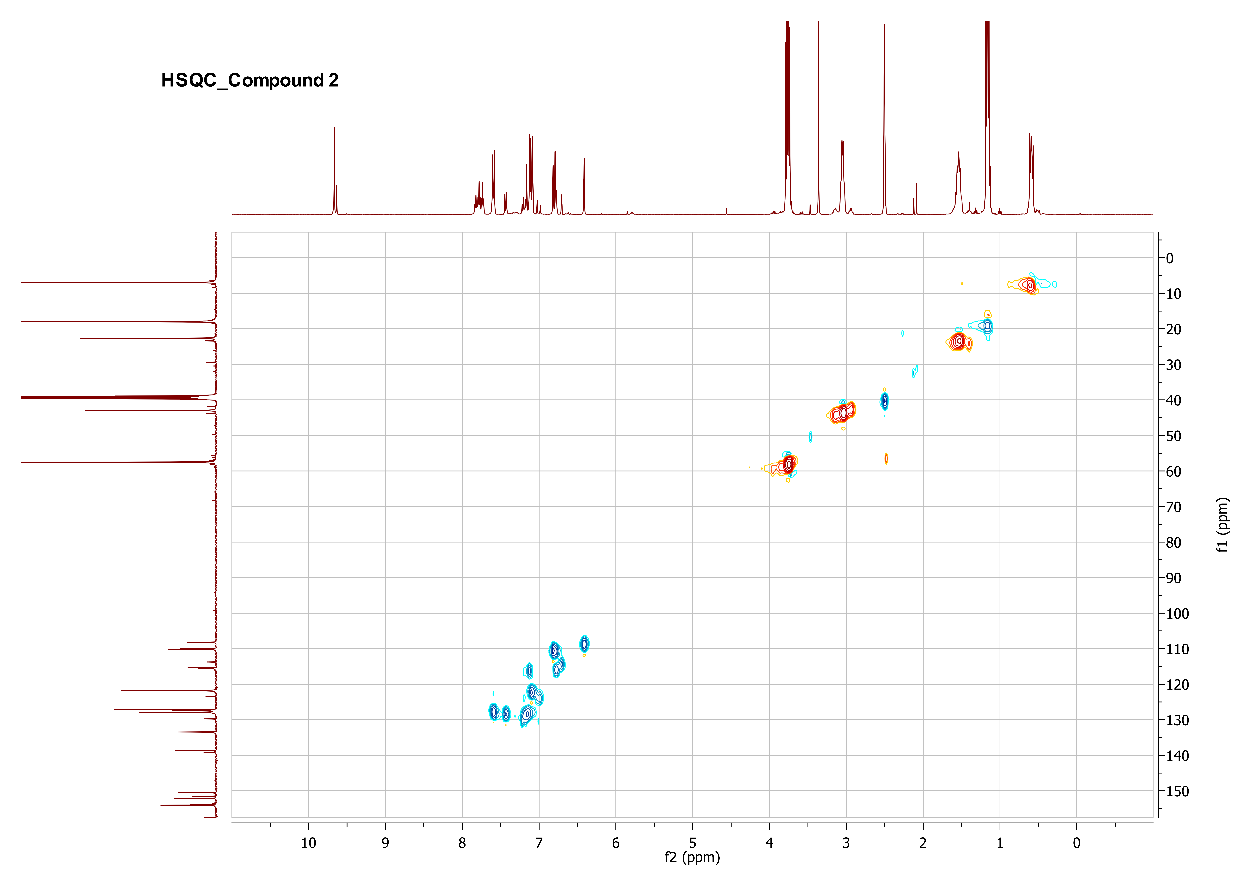


Figure S12: HSQC spectrum of compound 2


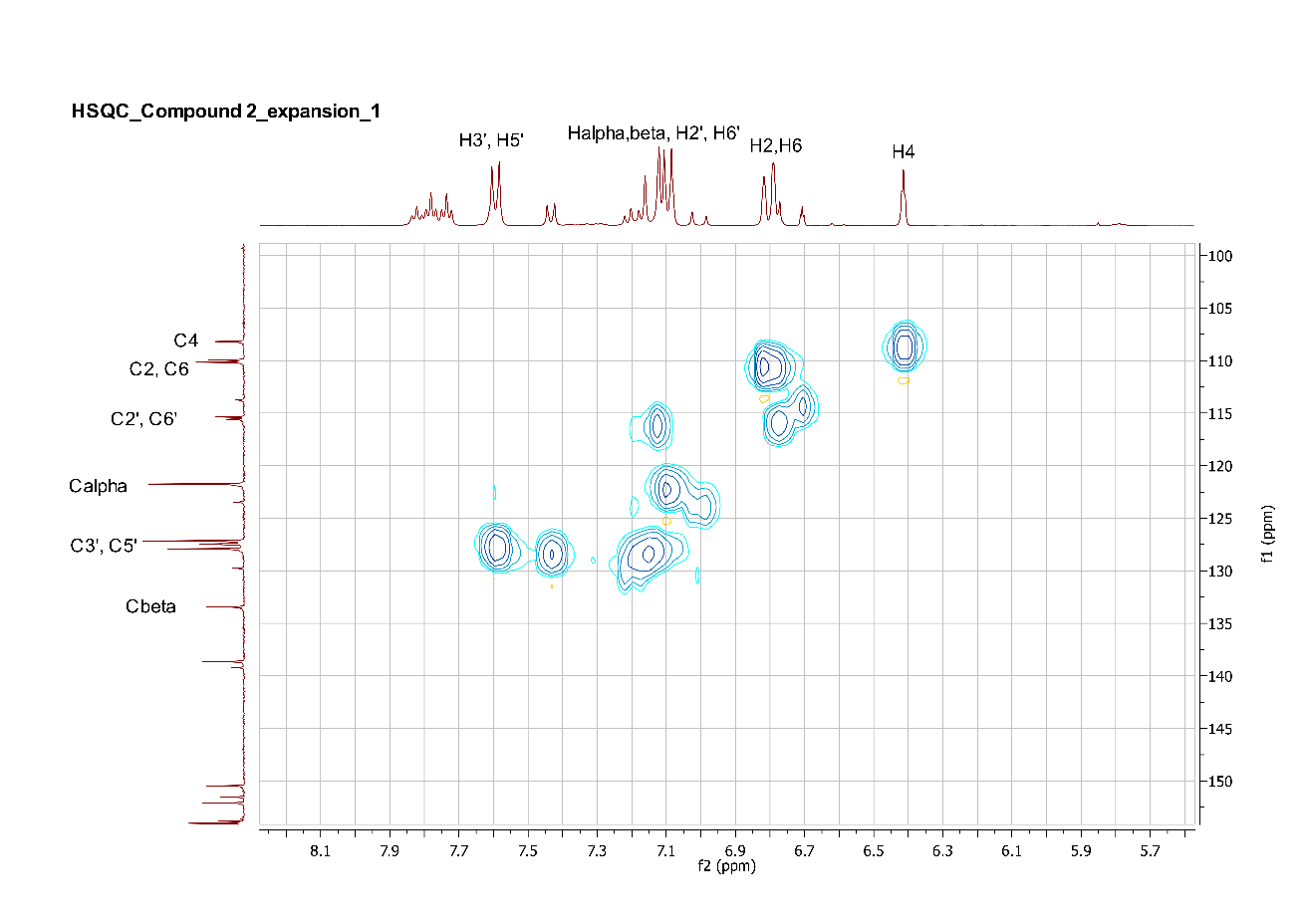


Figure S13: HSQC spectrum of compound 2, expansion 1


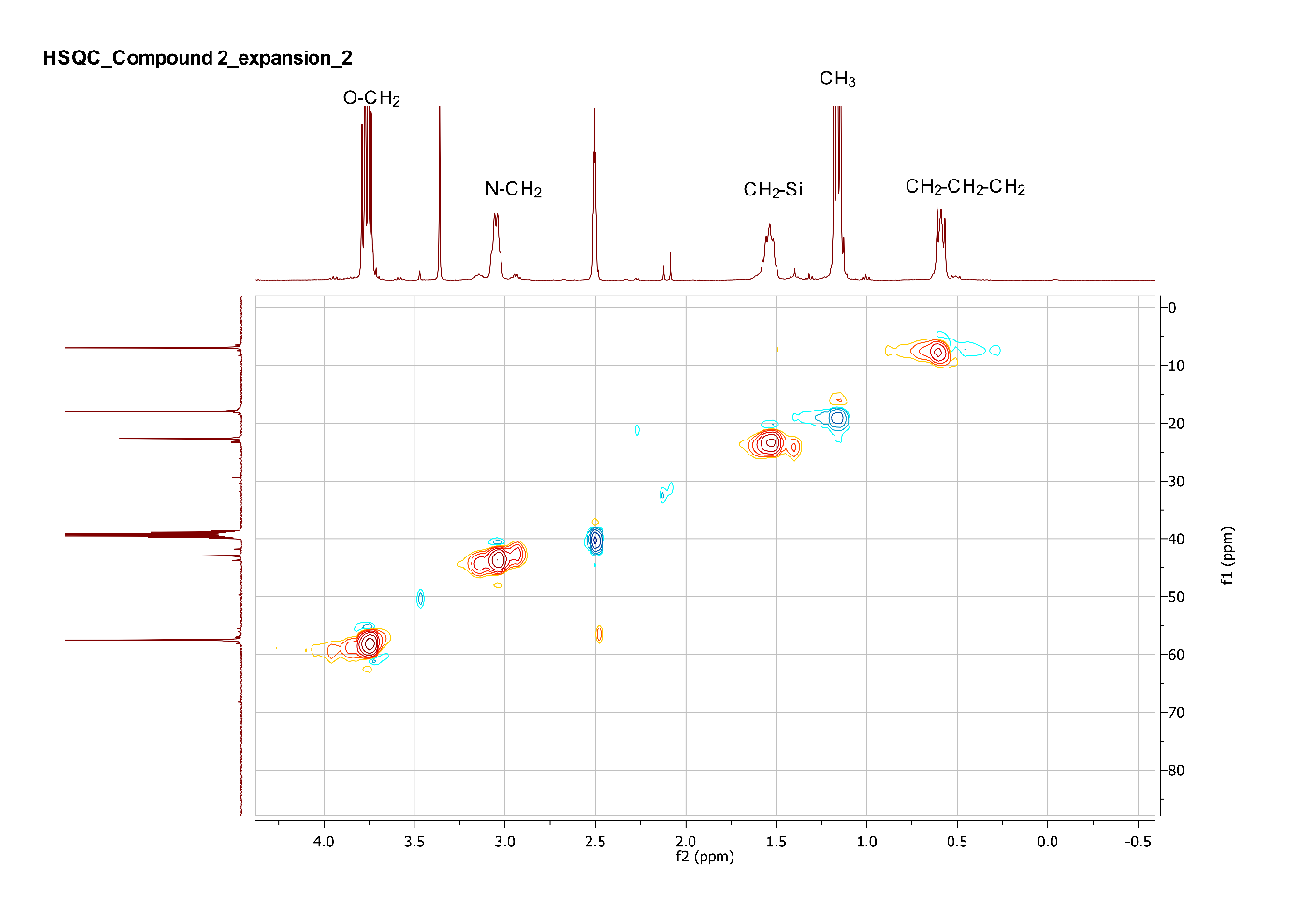


Figure S14: HSQC spectrum of compound 2, expansion 2


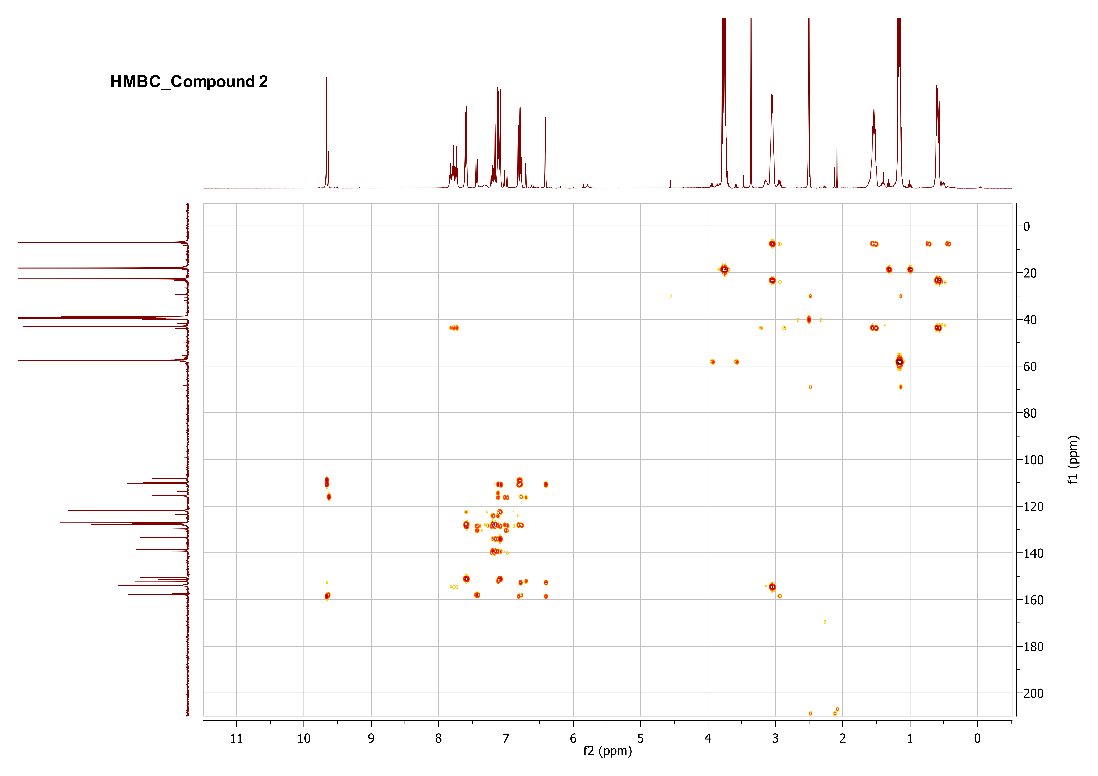


Figure S15: HMBC spectrum of compound 2


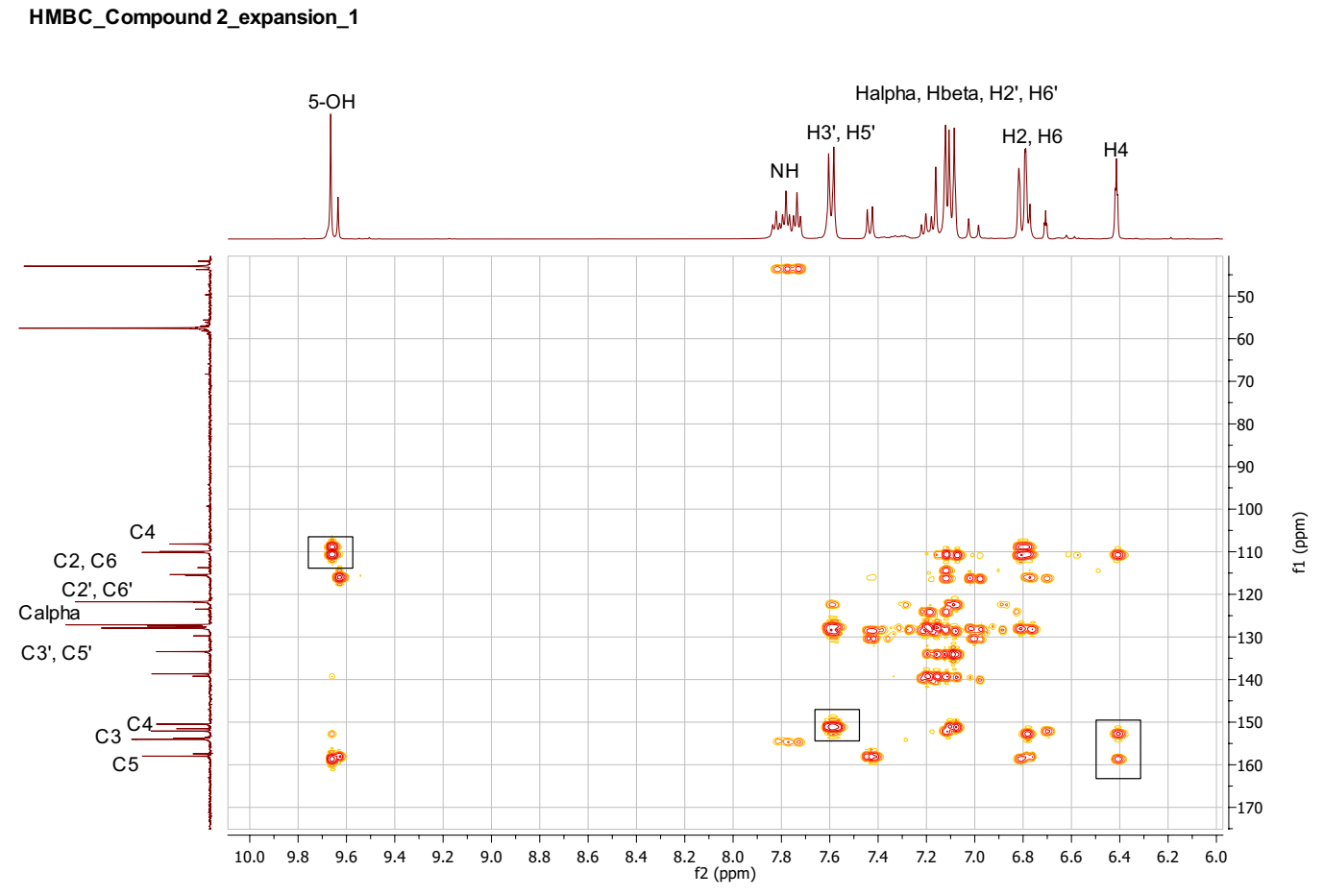


Figure S16: HMBC spectrum of compound 2, expansion 1


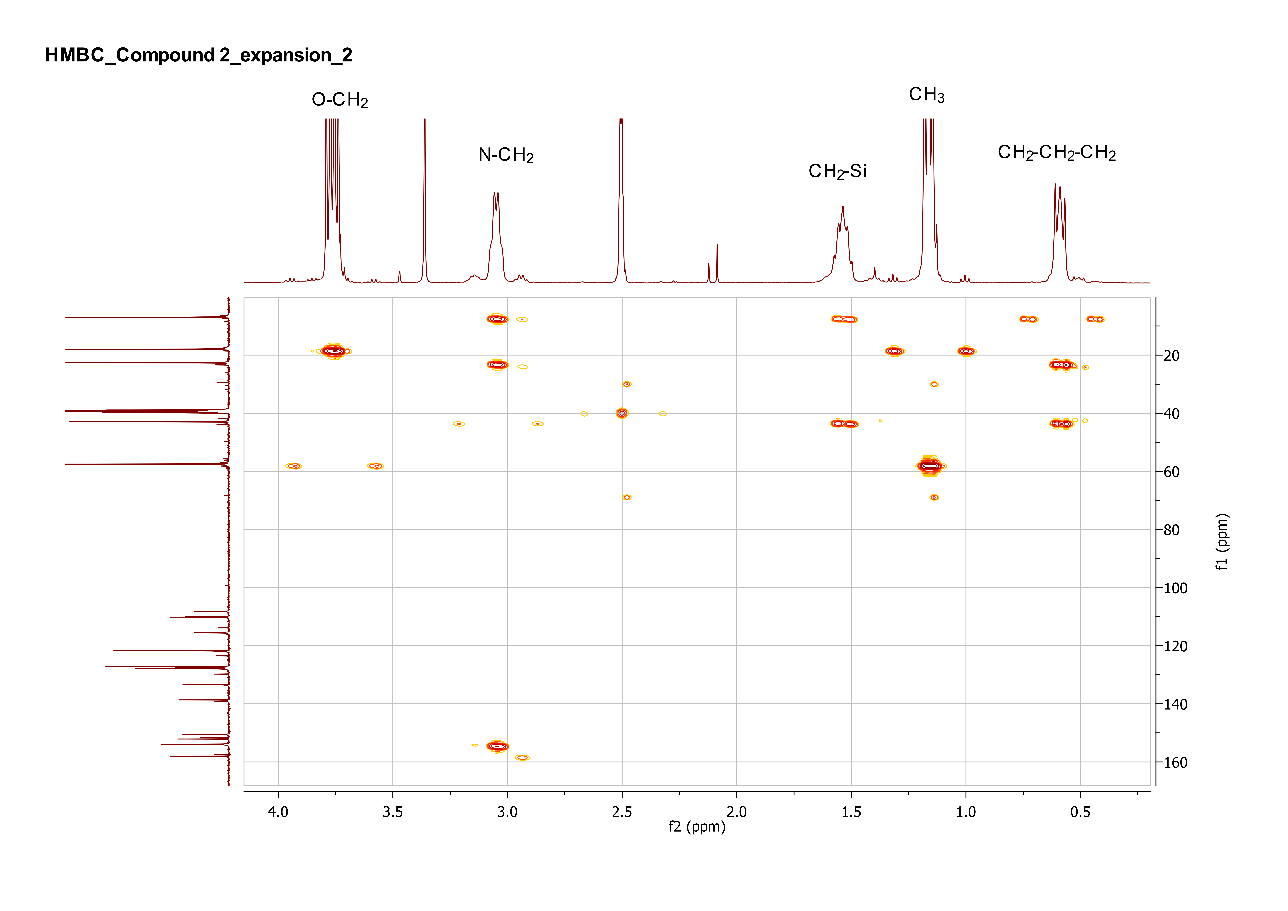


Figure S17: HMBC spectrum of compound 2, expansion 2


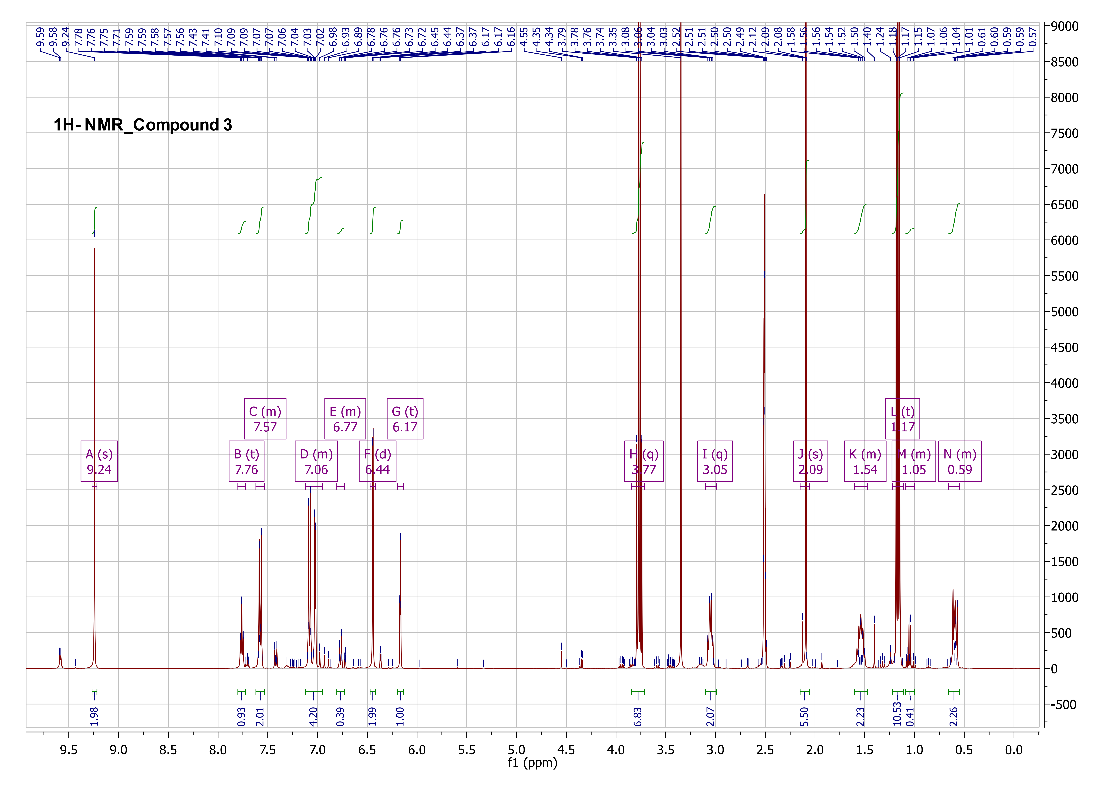


Figure S18: ^1^H-NMR spectrum of compound 3


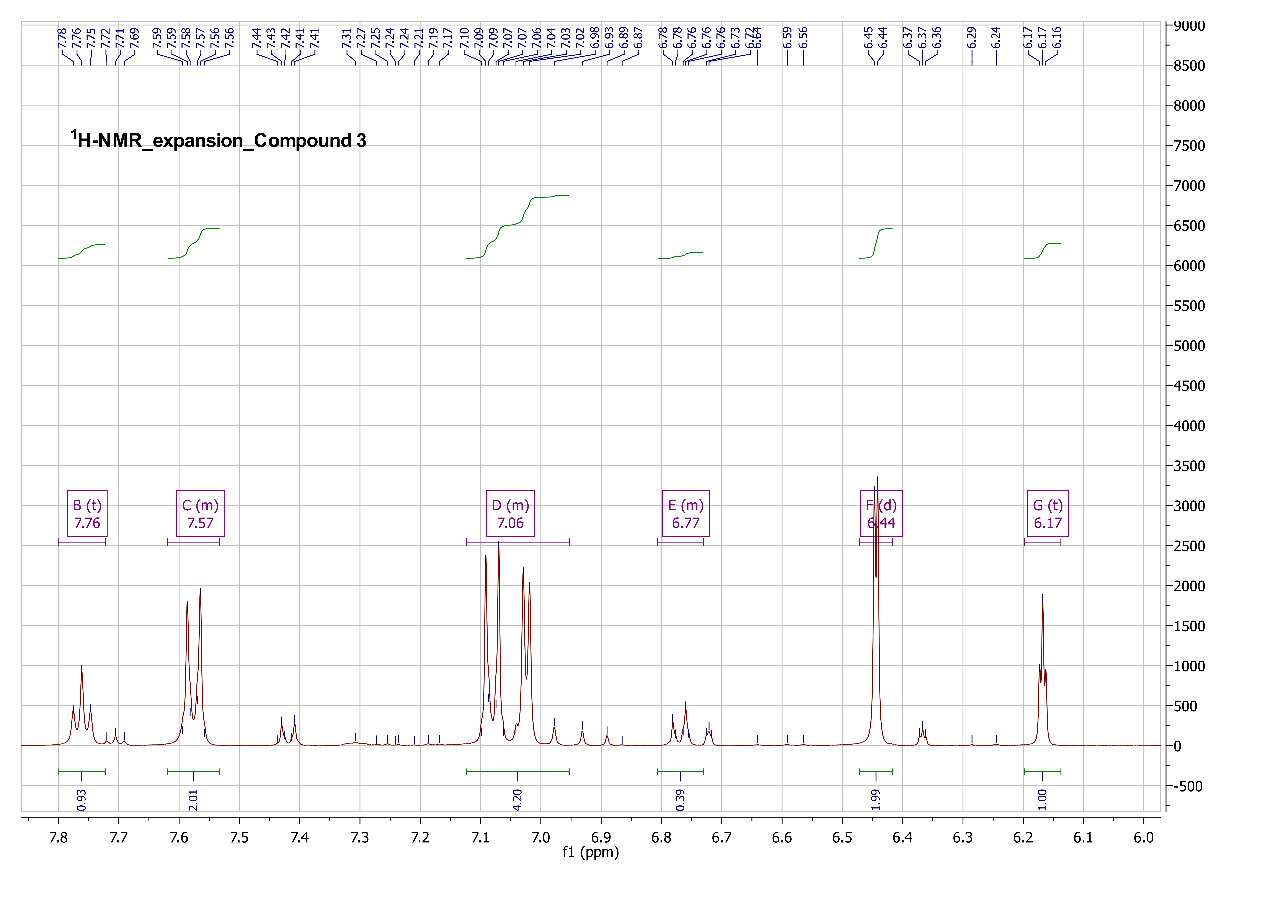


Figure S19: ^1^H-NMR spectrum of compound 3


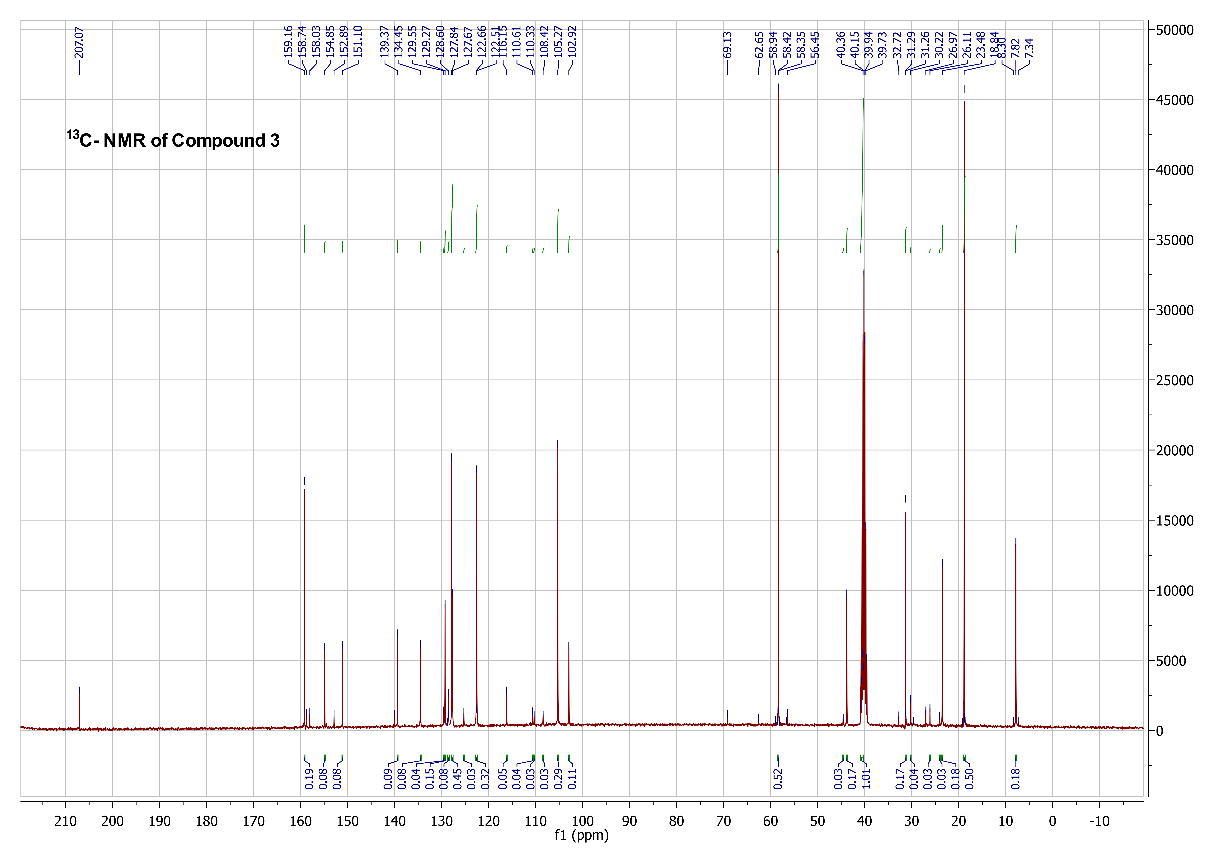


Figure S20: ^13^C-NMR spectrum of compound 3


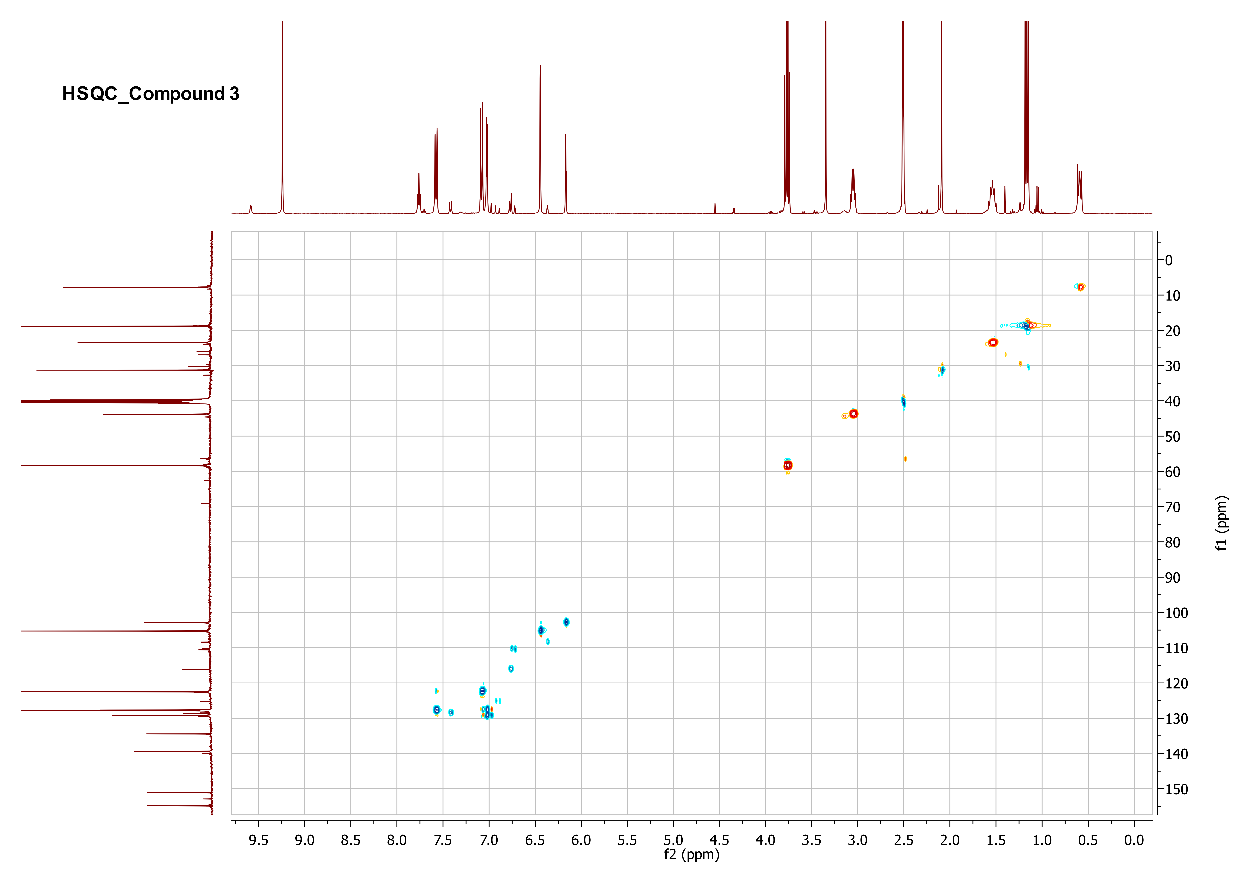


Figure S21: HSQC spectrum of compound 3


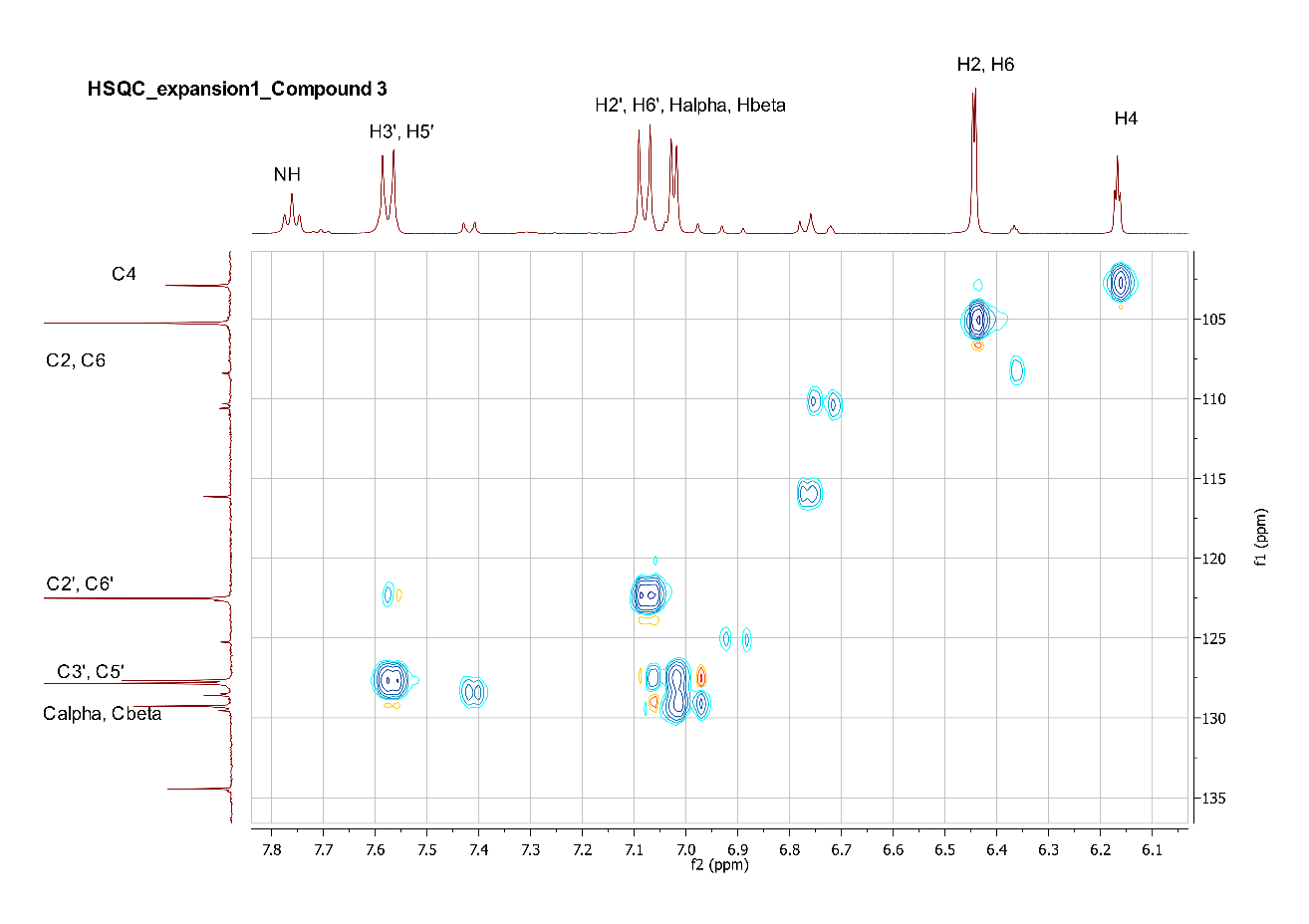


Figure S22: HSQC spectrum of compound 1, expansion 3


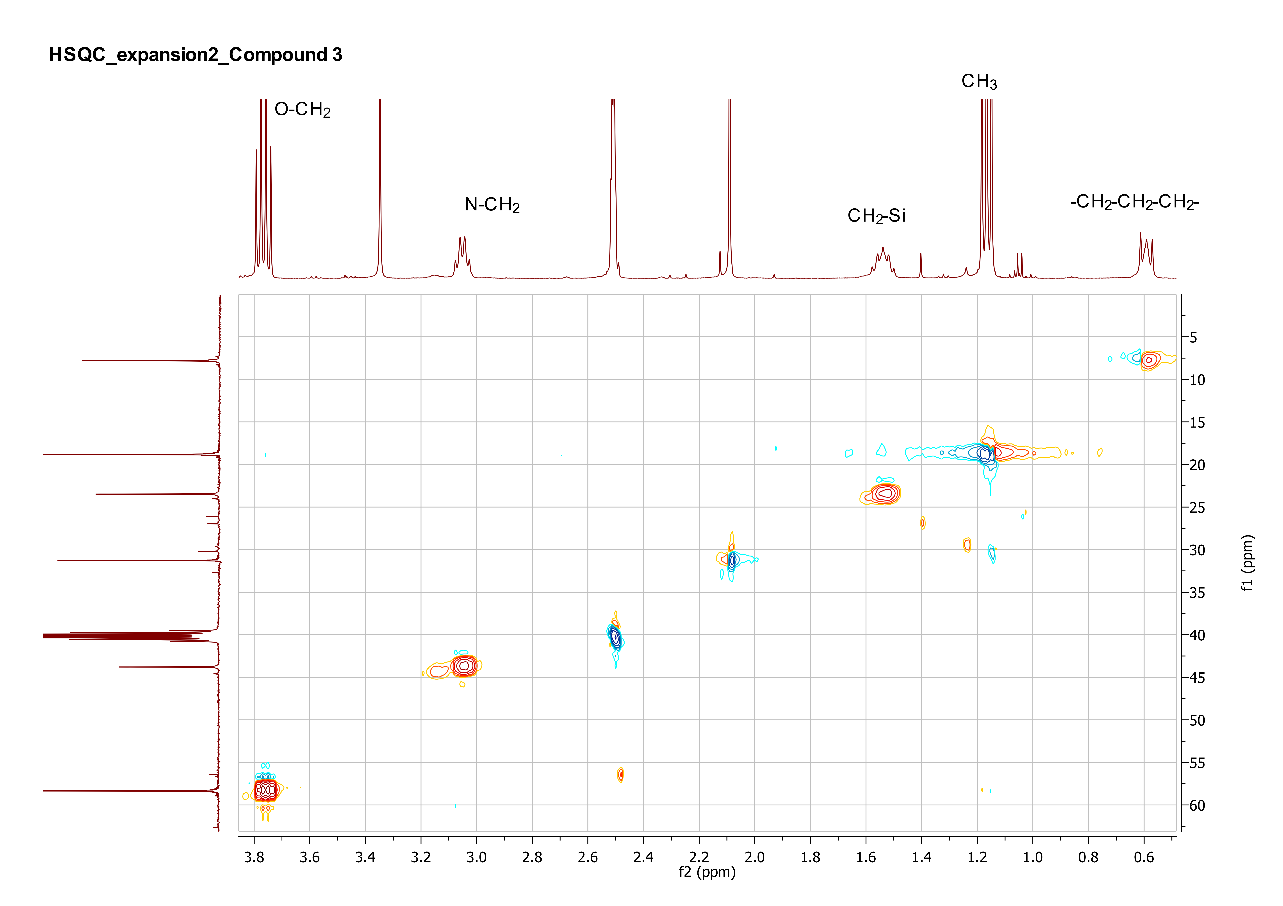


Figure S23: HSQC spectrum of compound 3, expansion 2


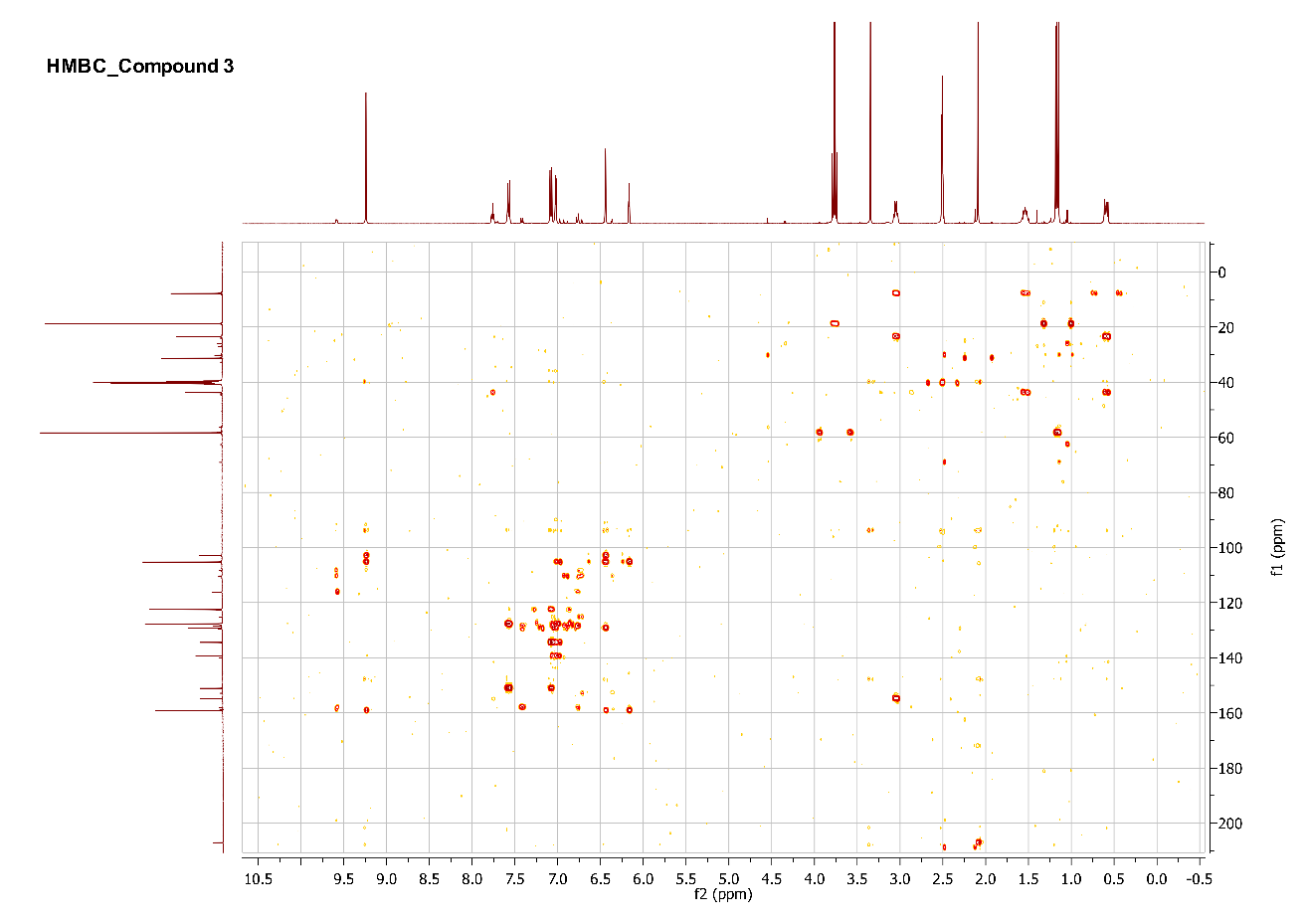


Figure S24: HMBC spectrum of compound 3


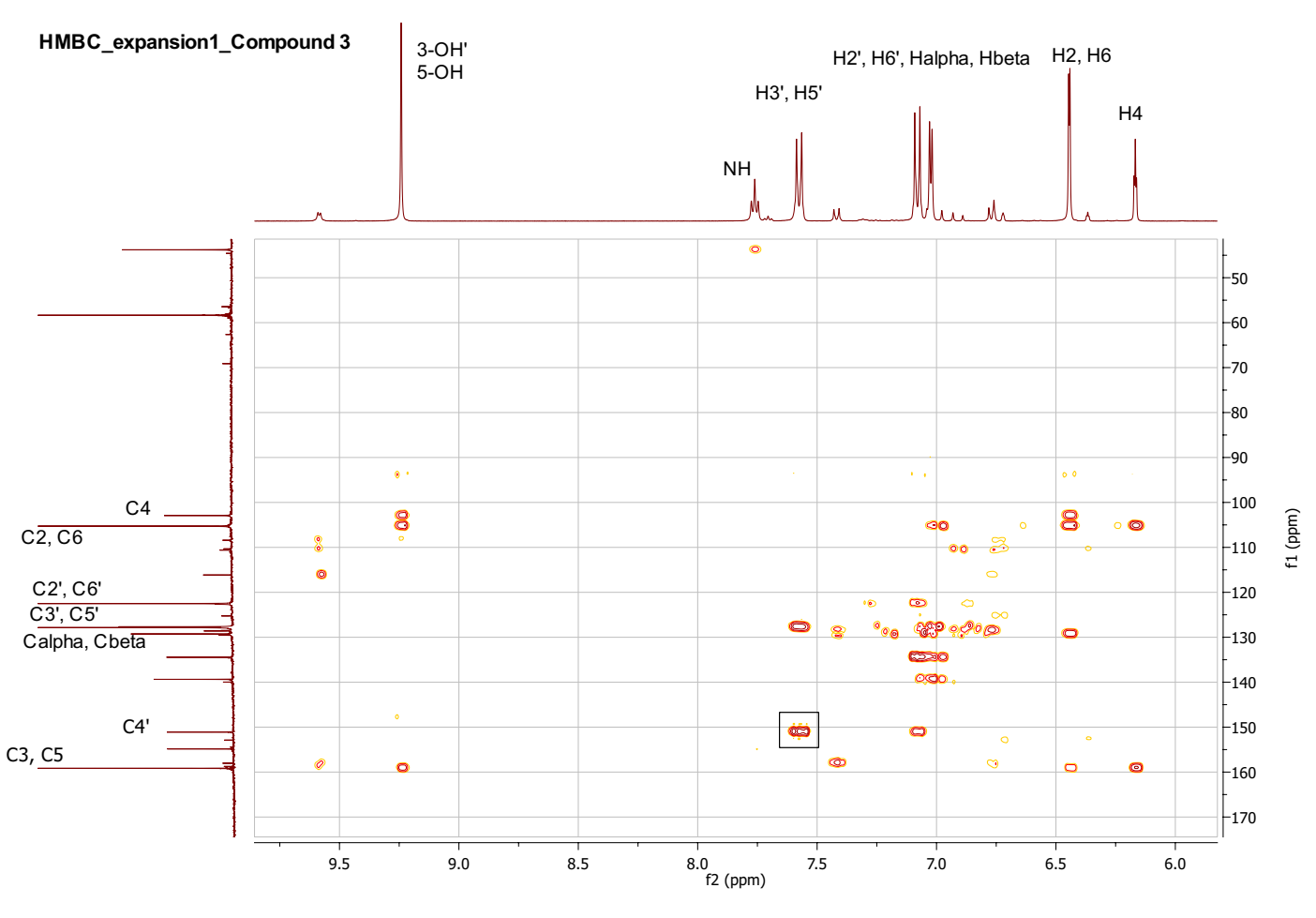


Figure S25: HMBC spectrum of compound 3, expansion 1


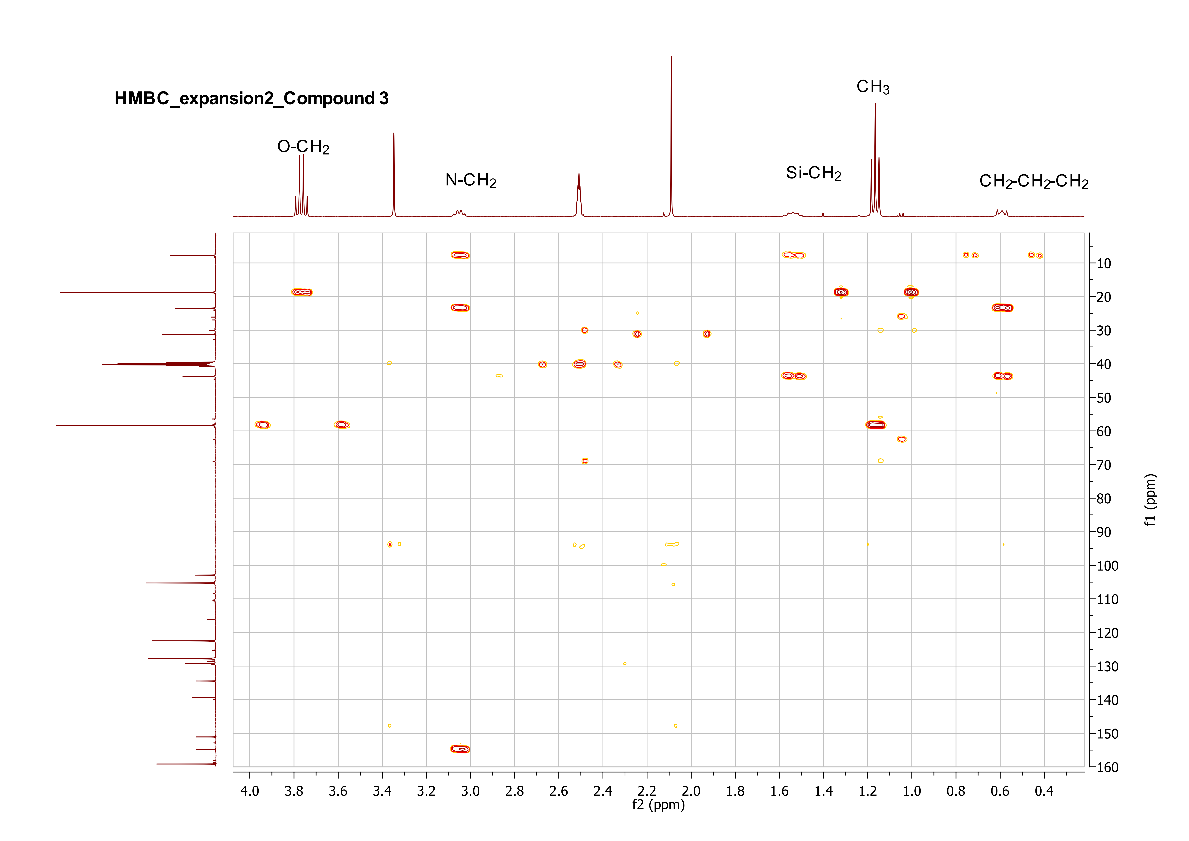


**Figure S26**: HMBC spectrum of compound 3, expansion 2

***Part 5. Mass spectroscopy of compound*** ***1, 2, and*** ***3****.*


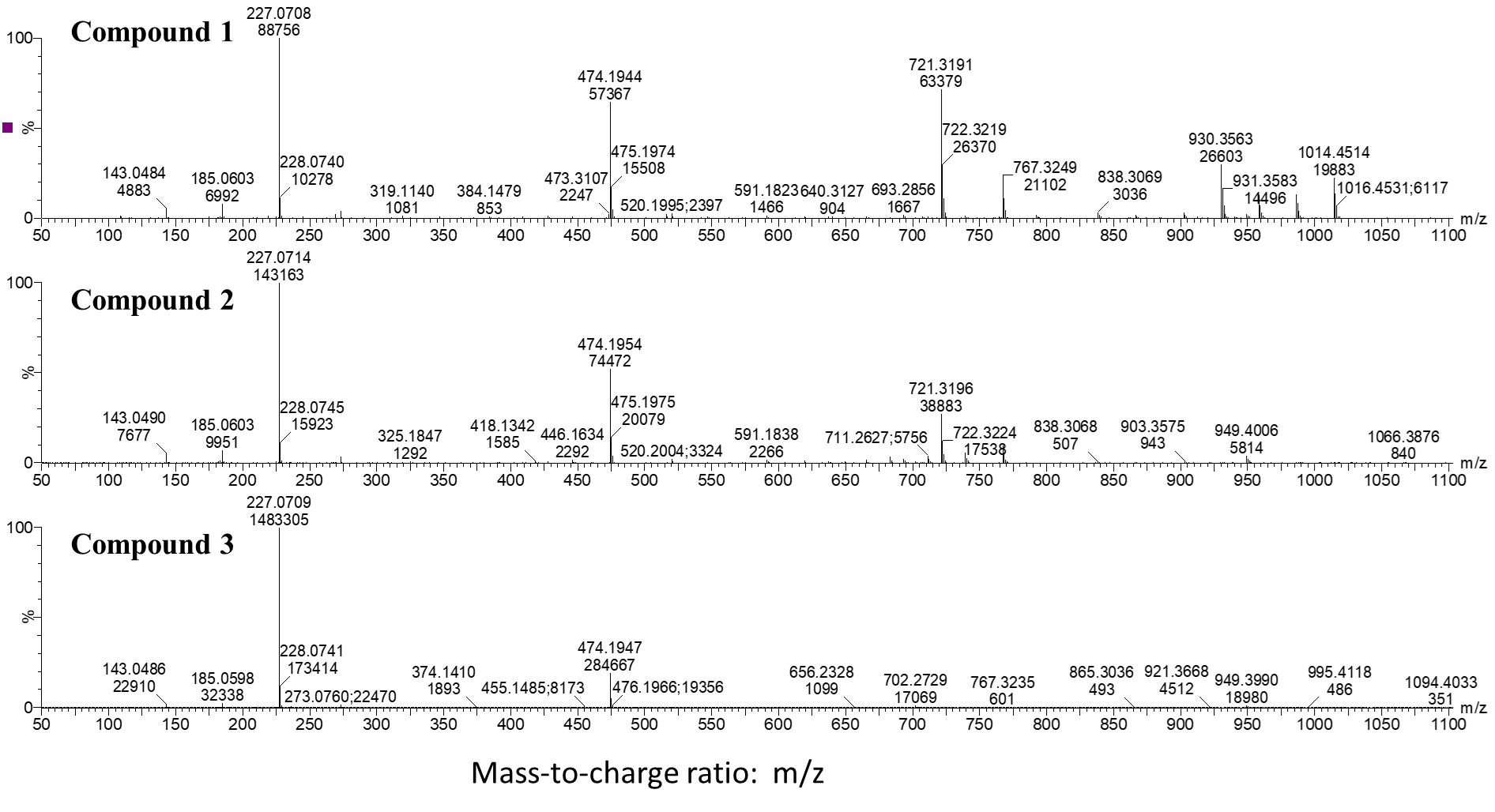


**Figure S27. Mass spectroscopy of compound** **1, 2, and** **3**.


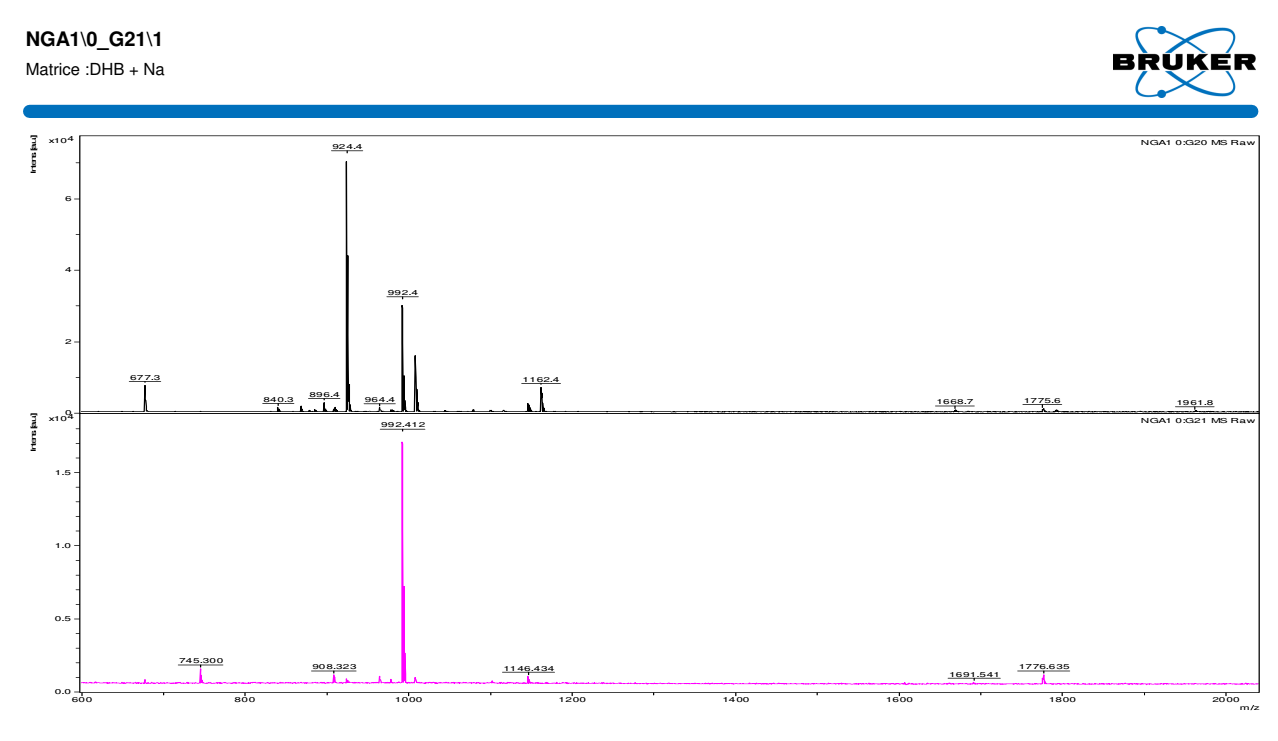


**Figure S28. MALDI-TOF Compound** **1**


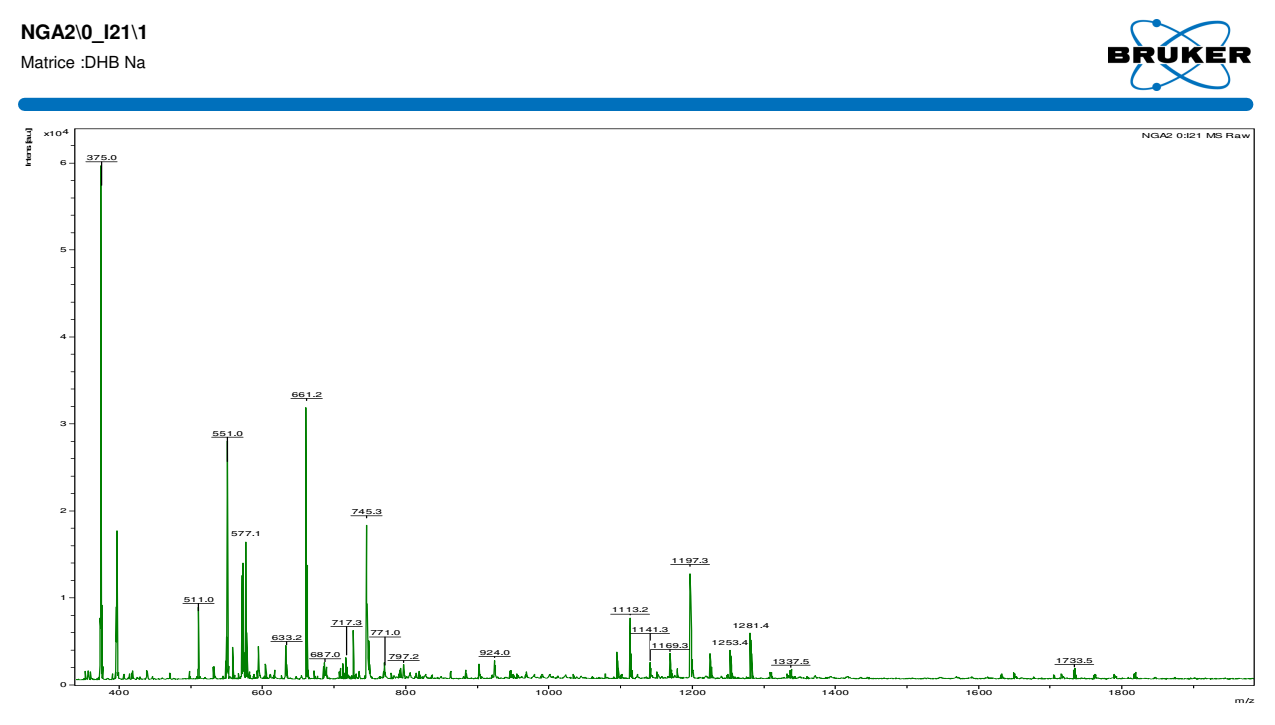


**Figure S29. MALDI-TOF Compound** **2**


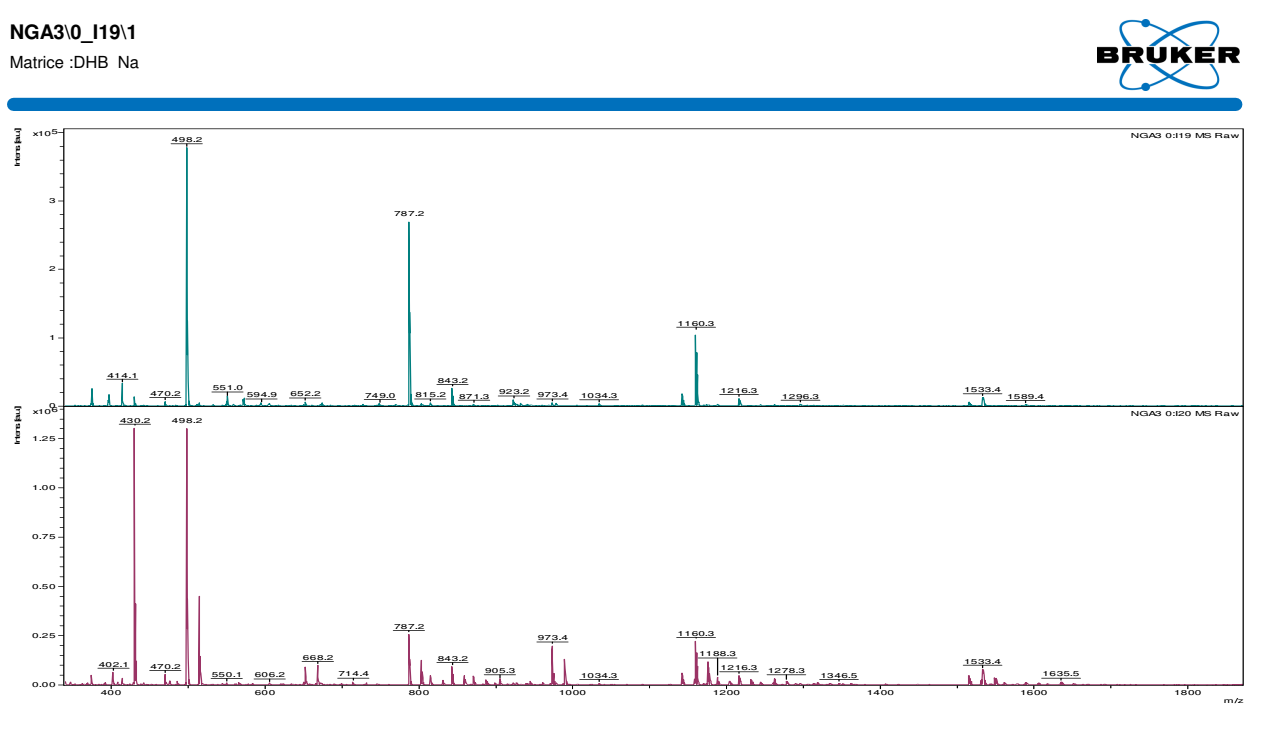


**Figure S30. MALDI-TOF Compound** **3**

**Part 6. Application of DLVO and XDLVO models between two spherical nanoparticles**

1. **Description of models**

Nanoparticle aggregation is mainly governed by a very weak attraction at large distances, an electrostatic repulsion at intermediate distances, and a strong attraction at short distances (1). These interaction energies are described in the classical DLVO (Derjaguin – Landau –Verwey - Overbeek) theory, describing the balance between the van Der Waals attraction and the electrostatic double layer interactions, which can be either attractive or repulsive depending on the surface charge of the two nanoparticles. The DLVO theory has been successfully and widely used as a theoretical model to assess quantitatively aggregation as a function of separation distance between the interacting nanoparticles (2). However, in these classical DLVO theory, the colloidal particle surfaces are assumed to be chemically inert, ignoring interaction energy due to polar nature of silanol at the silica interfaces and their proton exchange with water. In an extended XDLVO theory, Van Oss *et al*. (3-5) added the short-range Lewis acid–base (AB) interactions component to account for polar interactions at the interface, and partially explained the discrepancies between the DLVO predictions and experiment results.

Based on the classical DLVO theory, the total interaction energy (ɸ_Tot_) between charged surfaces in a liquid medium is obtained by sum of the Lifshitz–van der Waals energy (ɸ_vdW_), the electrostatic energy (ɸ_DL_), the Born energy (ɸ_Born_).

$\Phi_{DLVO}\left( h \right)=\Phi_{vdW}\left( h \right)+\Phi_{dl}\left( h \right)+\Phi_{Born}\left( h \right)$ Equation S1

In the extended XDLVO theory, the acid–base interaction energy (ɸ_AB_) is added.

$\Phi_{XDLVO}\left( h \right)=\Phi_{vdW}\left( h \right)+\Phi_{dl}\left( h \right)+\Phi_{Born}\left( h \right)+\Phi_{AB}\left( h \right)$ Equation S2

From a thermodynamic standpoint, attraction or adhesion occurs when ɸ is negative whereas repulsion occurs when ɸ is positive.

The Lifshitz–Van der Waals potential energy exerted an attractive force and has a negative sign. We adopted the equation for two spherical particles established by Feke *et al*. (6) and used by Ryan and Gschwend (7).

$\Phi_{vdW}\left( h \right)=\frac{A_{123}}{12}\left( \frac{R_{p}}{\xi^{2}+{\xi R}_{p}+\xi}+\frac{R_{p}}{\xi^{2}+{\xi R}_{p}+\xi+R_{p}}+2 Ln \left[ \frac{\xi^{2}+{\xi R}_{p}+\xi}{\xi^{2}+{\xi R}_{p}+\xi+R_{p}} \right] \right)$ Equation S3

with $R_{p}=\frac{r_{p2}}{r_{p1}}$ Equation S4

and $\xi=\frac{h+r_{p1}+r_{p2}}{{2 r}_{p1}}$ Equation S5

In the case of silica nanoparticles with anionic surface charges, the electrostatic potential energy (ɸ_DL_) resulting from the electrostatic double layer of ions around sphere 1 and sphere 2, which exerts a repulsive force and has a positive sign. We adopted the formula of Hogg *et al.* (8).

$\Phi_{dl}\left( h \right)= \pi\varepsilon_{r} \varepsilon_{0}\frac{r_{p1} r_{p2}}{\left( r_{p1}+r_{p2} \right)}\left[ 2 \psi_{p_{1}}\psi_{p_{2}} \ln\left( \frac{1+e^{- к h}}{1-e^{- к h}} \right)+ \left( \psi_{p_{1}}^{2}+\psi_{p_{2}}^{2} \right) \ln\left( 1-e^{-2 к h} \right) \right]$ Equation S6

with the Debye-Hückel reciprocal length к

$к= \sqrt{\frac{2 I_{s} N_{A} 1000 e^{2}}{\varepsilon_{r} \varepsilon_{0}k_{B} T}}$ Equation S7

In our experimental conditions, the ionic strength is low and the surface potential decays exponentially with distance. The Stern layer effect is considered negligible and as carried out in previous studies (9-12), we assigned to the electric potential of the nanoparticle surface (ψ_s_) the zeta potential (ζ) value that we measured using electrophoretic mobility at each pH.

At very short distance (0.1 nm), the Born energy generated very strong repulsion force. They result from the impossibility of covering the electronic clouds and have the role of preventing too close contact between the particles. We adopted the formula established by Feke *et al*. (6) and Ryan & Gschwend (7).

$\Phi_{Born}\left( h \right)= \frac{A_{123}}{75600 \xi} \left( \frac{\sigma_{Born}}{r_{p1}} \right)^{6} \left[ \begin{aligned} \frac{-4 \xi^{2}-14\left( R_{p}-1 \right) \xi-6 \left( {R_{p}}^{2}-7 R_{p}+1 \right)}{\left( 2 \xi-1 +R_{p} \right)^{7}} \\ + \frac{-4 \xi^{2}+14\left( R_{p}-1 \right) \xi-6 \left( {R_{p}}^{2}-7 R_{p}+1 \right)}{\left( 2 \xi+1 +R_{p} \right)^{7}} \\ +\frac{4 \xi^{2}+14\left( R_{p}-1 \right) \xi+6 \left( {R_{p}}^{2}+7 R_{p}+1 \right)}{\left( 2 \xi+1 +R_{p} \right)^{7}} \\ +\frac{4 \xi^{2}-14\left( R_{p}-1 \right) \xi+6 \left( {R_{p}}^{2}+7 R_{p}+1 \right)}{\left( 2 \xi-1 +R_{p} \right)^{7}} \end{aligned} \right]$ Equation S8

The Lewis acid-base potential, ɸ_𝐴B_, describes polar interactions between surfaces. ɸ_𝐴B_ between two spheres was established by Oss (13).

$\Phi_{AB}\left( h \right)= 2 \pi\frac{r_{p1} r_{p2}}{\left( r_{p1}+r_{p2} \right)}{{\lambda_{AB} \Phi}_{AB\left( h=h_{0} \right)} e}^{\left( \frac{h_{0}-h}{\lambda_{AB}} \right)}$ Equation S9

Where λ_AB_ is the energy decay length, ɸ_ΑΒ_ _(h=h0)_ is the energy at minimum separation distance, h is the separation distance, h_0_ is the minimum separation and generally accepted to be 0.157 nm. In the case of silica, since ɸ_ΑΒ_ _(h=h0)_ is negative sign, ɸ_𝐴B_ is an attractive energy potential.

Finally, when a magnetic field is applied, superparamagnetic nanoparticles become align to it. The maximum magnetic attraction force occurs when particle dipoles are oriented in a head-to-tail configuration and then both magnetic moments are parallel. The magnetic interactions are described by a dipole–dipole interaction potential. The maximum magnetic interaction energy (ɸ_M_) is expressed with the formula used by Viota *et al*. (14) and Phenrat *et al*. (15). ɸ_M_ is negative sign and exerted an attractive force.

$V_{M} = - \frac{8 \pi}{9} \frac{\mu_{0} M^{2}a^{3}}{\left( \frac{s}{a}+2 \right)^{3}}$ Equation S10

The mean inter-particle distance (IPD) can be assessed from the equation of Hao and Riman (2006):

$IPD=2 r \left[ \left( \frac{{PPF}_{m}}{PPF} \right)^{\frac{1}{3}}-1 \right] for PPF< {PPF}_{m}$ Equation S11

with the maximum particle packing fraction (PPF_m_) of hexagonally closed packed spheres or of face centered close packed spheres network defined as:

${PPF}_{m}= \pi\frac{\sqrt{2}}{24} \approx0.52359$ Equation S12

Surface charge density of nanoparticles was calculated from the electrokinetic results according to the formula proposed by Montagne *et al.* (19) and Shi *et al.* (20), corresponding to the Poisson-Boltzmann equation describing the charge-potential relationship for a sphere.

$\sigma= \varepsilon_{r} \varepsilon_{0}\frac{k_{B} T}{\left( z+e \right)} к \left[ 2 sinh\left( \frac{z \psi}{2} \right)+\frac{4}{к a}\tanh\left( \frac{z \psi}{4} \right) \right]$ Equation S13

Where z is the valence of the ionic species, ψ is the surface electrostatic potential, a is the radius of the spherical nanoparticle.

1. **Simulations**

Since *trans*-resveratrol grafted to nanoparticles is fragile molecule, expensive, available pure in very small quantities, it is not possible to carry out the complete physico-chemical characterization required to apply DLVO and XDLVO models, which would require the consumption of large quantities of grafted nanoparticles. Therefore, simulations of this work were carried out using parameters reported in **Table S2**, which originate from aggregation studies of silica nanoparticles already published.


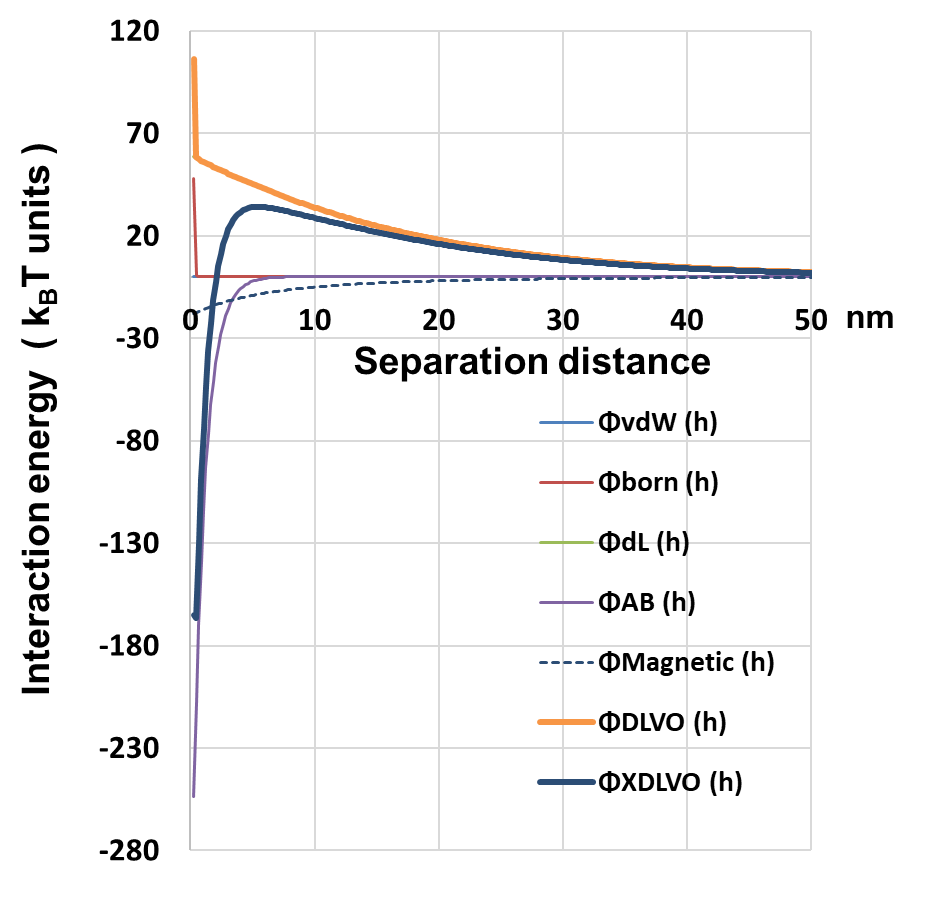


**Figure S31**: Interaction energy of two spherical nanoparticles (50 µg/mL, volume fraction 2.25 x 10^-5^) in aqueous TRIS buffer calculated using DLVO and XDLVO models with parameters reported in **Table S2**, assuming the surface electric potential equal to the experimental zeta potential. Interaction energy is expressed in k_B_T units.

The electrostatic energy potential (ɸ_DL_) of the double layer of counter-ions around each nanoparticle is positive sign and exert a repulsive force. ɸ_DL_ mainly increases with zeta potential, and therefore mainly increases with pH value. It is supplemented by the Born energy potential ɸ_Born_, but only within the first 0.10 nm. The sum ɸ_DL_ + ɸ_Born_ is balanced by the attractive van der Waals energy potential (ɸ_vdW_) of negative sign, which is dominant at short distance and become negligible for h > 30 nm. When Lewis acid-base potential ɸ_𝐴B_ is introduced in the model to take into account polar silanols of the silica surface, ɸ_XDLVO_ strongly decreases compared to ɸ_DLVO_. The acid–base contribution of ɸ_𝐴B_ is more predominant from 8 to 1 nm inter-distance and mainly decreases the first maximum of the ɸ_XDLVO_ curve, corresponding to the binding energy barrier. In acidic condition, this first maximum disappeared of the ɸ_XDLVO_ curve (Figure S2). Finally, ɸ_Magnetic_ observed in Figure S1 has a reduced aggregation contribution due to the reduce size of magnetite core.


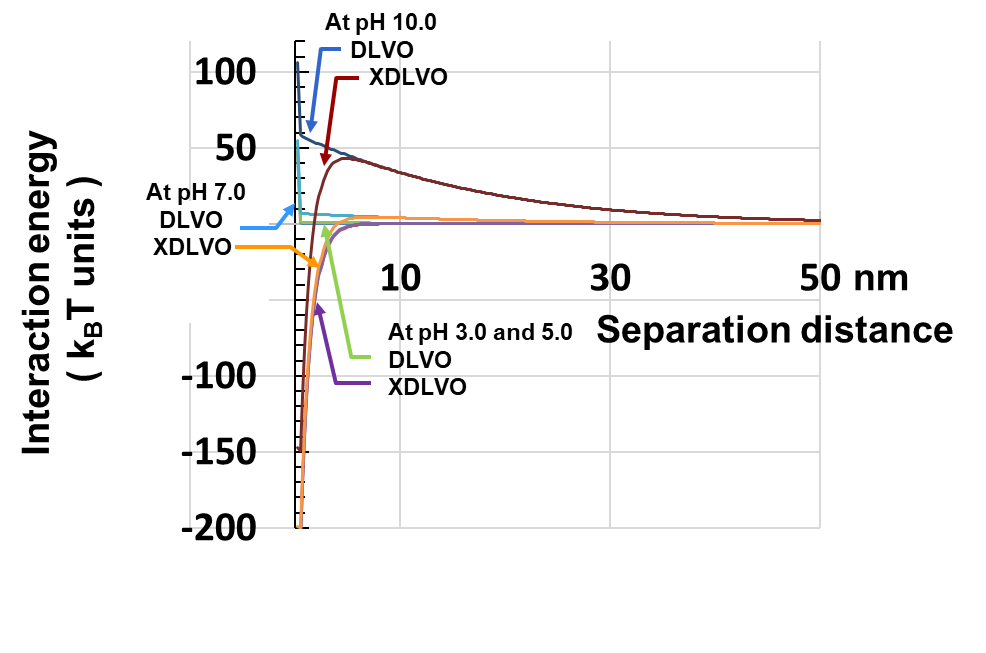


**Figure S32**: Potential energy of interaction of two spherical nanoparticles is expressed in k_B_T unit as a function of the distance (h) between the two surfaces of spherical nanoparticles (50 µg/mL, volume fraction 2.25 x 10^-5^) in aqueous TRIS buffer, calculated with parameters reported in **Table S2**. Interaction energy is expressed in k_B_T units.

A colloidal suspension is typically considered as stable if the total interaction energy develops a positive energy barrier larger than 15 k_B_T units (16). We observe that this energy barrier increases with shell diameter in a reduced proportion for pH below 9.0, and it increases very strongly only for a pH above 10.0. The energetic barrier increases from 15 to 31 kBT between 25 nm and 50 nm shell radius at pH 10.0. Thanks to ɸ_DL_ increase, the energy barrier went up with the rising pH (Figure S33). For our core-shell nanoparticles with an average diameter of 93 nm, we observe that the DLVO model always predicts an energy barrier greater than 15 k_B_T units in all cases (CS, CS1, CS2, CS3). DLVO predictions does not correspond to aggregation behavior observed by DLS experiments (Figure 3). By contrast, the XDLVO model predicts very reduced energy barrier values below pH 9.0. It predicts that charge repulsion is enough to prevent CS aggregation at pH 9.0 for functionalized CS1, CS2 and CS3 nanoparticles, but not for non-functionalized CS ones, which need to reach pH 10.0 for colloidal stability. These predictions conformed with DLS results.


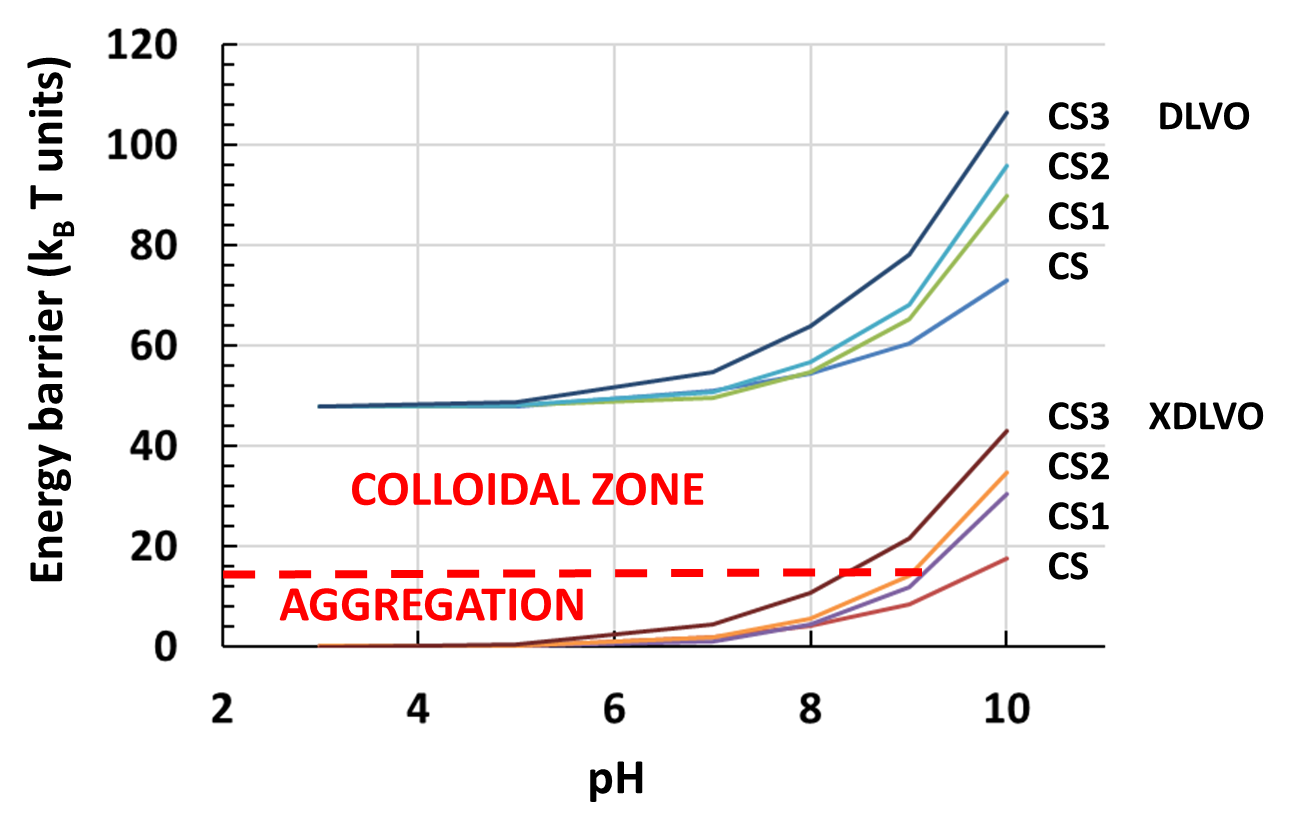


**Figure S33**: Energy barrier of colloidal suspension of nanoparticles (50 µg/mL, volume fraction 2.252 x 10^-5^) in aqueous TRIS buffer, calculated using DLVO and XDLVO models with parameters of **Table S2**, assuming the surface electric potential equal to the experimental zeta potential. Energy barrier is expressed in k_B_T units.

**Magnetic properties influence on colloidal stability**

Using the nanoparticle volume fraction (Vf = 2.252 x 10^-5^), we deduced the value of the mean inter-particle distance to be of 2561 nm, which corresponds to less than 11 nanoparticle diameters. At this distance, the magnetic interaction can roughly be estimated to be much less than thermal energy (ɸ_M_(2561 nm) < 10^-2^ k_B_T). Consequently, a random orientation of the magnetic moments in the absence of external field can be assumed (Wagner et Fisher 2006), and the mean magnetic interaction equal zero under this assumption. In the absence of an applied magnetic field, core/shell nanoparticles behave as non-magnetic particles. This result explains why aqueous suspension of CS conserves its colloidal behavior more than one day at laboratory temperature. This behavior was previously highlighted by Wagner & Fisher (17)and Leong *et al*. (18).

According to figure 1, saturation magnetization of magnetite core and core/shell nanoparticles are 8.0 and 2.0 emu/g, respectively. When the magnetic dipoles are aligned parallel to the magnetic external field, the attractive magnetic force can be assessed using formula used by Calderon *et al*. (19). It decreases according to the inverse of the forth power of the separation distance.

$F_{m}\left( d \right)= - \frac{1.202}{2 \pi\mu_{0}} \frac{{3 m}^{2}}{d^{4}}$ Equation S14

When two core/shell composite nanoparticles are in contact, the two magnetic cores are separated by 2 * (46.5 – 9 ) = 70 nm of non-magnetic silica thickness. Then, we deduce from the formula S13 that the attractive magnetic force is divided by 712 compared with forces of two aggregating cores.

When a magnetic field is simulated (B = 0.050 T), for magnetite core of 18 nm diameter, magnetic moment is high enough to exert magnetic attraction which dominates the interaction energy, and there is no energy barrier to resist aggregation. And there is no longer a predicted energy barrier to aggregation. These results are in accordance with those of Phenrat *et al*. (15). We use a magnet to wash magnetic nanoparticles and to change their liquid medium.

**Surface charge density of functionalized nanoparticles**


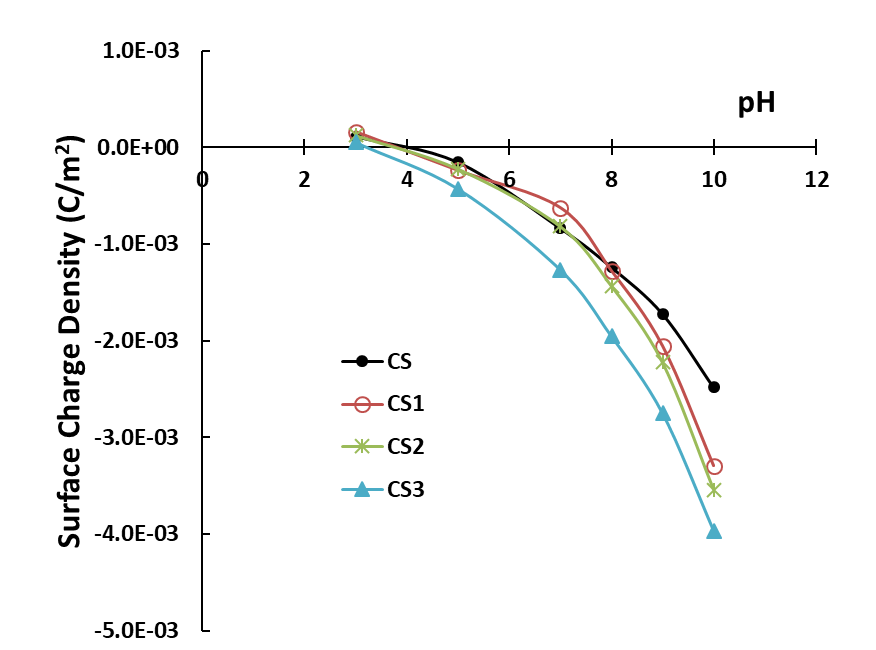


**Figure S34**: Surface charge density of silica nanoparticles (50 µg/mL, volume fraction 2.252 x 10^-5^), calculated using **equation S13** and parameters of **Table S2**, assuming the surface electric potential equal to the experimental zeta potential.

Since *trans*-resveratrol grafted nanoparticles was produced in small amount, it is not possible to carry out the complete physico-chemical characterization required for assessing experimentally all parameters of DLVO and XDLVO models. All these simulations are only indicative since we use the rare values of parameters published for silica nanoparticles. We neglected steric forces of the silane-resveratrol links grafted at low density to silica surface. We hypothesis that TRIS-HCl buffer behaves as inert ions which do not adsorb on the colloidal surface. Furthermore, we do not add some non-DLVO additional forces occurring in aqueous media, such as short distance repulsive hydration forces, osmotic repulsion and elastic-steric repulsion (15).

**References**

1. Butt H-J, Kappl M. Surface and Interfacial Forces: Wiley‐VCH Verlag GmbH & Co. KGaA; 2010.

2. Zhang W. Nanoparticle Aggregation: Principles and Modeling. Advances in Experimental Medicine and Biology. 811: Springer, Dordrecht; 2014.

3. Oss CJ. The extended DLVO theory. Interface Science and Technology. 2008;16:31-48.

4. Oss CJV, Good RJ, Chaudhury MK. The role of van der Waals forces and hydrogen bonds in “hydrophobic interactions” between biopolymers and low energy surfaces. Journal of Colloid and Interface Science. 1986;111:378-90.

5. Oss CJV. Hydrophobicity of biosurfaces — Origin, quantitative determination and interaction energies. Colloids and Surfaces B: Biointerfaces. 1995;5:91-110.

6. Feke DL, Prabhu ND, Mann JJA, Mann III JA. A formulation of the short-range repulsion between spherical colloidal particles. The Journal of Physical Chemistry. 1984;88:5735-9.

7. Ryan JN, Gschwend PM. Effects of ionic strength and flow rate on colloid release: Relating kinetics to intersurface potential energy. Journal of Colloid and Interface Science. 1994;164:21-34.

8. Hogg R, Healy TW, Fuerstenau DW. Mutual coagulation of colloidal dispersions. Transactions of the Faraday Society. 1966;62:1638-51.

9. Yeh L-H, Xue S, Joo SW, Qian S, Hsu J-P. Field effect control of surface charge property and electroosmotic flow in nanofluidics. The Journal of Physical Chemistry C. 2012;116:4209-16.

10. Atalay S, Ma Y, Qian S. Analytical model for charge properties of silica particles. Journal of Colloid and Interface Science. 2014;425:128-30.

11. Barisik M, Atalay S, Beskok A, Qian S. Size dependent surface charge properties of silica nanoparticles. The Journal of Physical Chemistry C. 2014;118:1836-42.

12. Yu Y, Ma L, Xu H, Sun X, Zhang Z, Ye G. DLVO theoretical analyses between montmorillonite and fine coal under different pH and divalent cations. Powder Technology. 2018;330:147-51.

13. Oss CJV. Interfacial Forces in Aqueous Media: Marcel Dekker, New York 2006.

14. Viota JL, Vicente JD, Durán JDG, Delgado AV. Stabilization of magnetorheological suspensions by polyacrylic acid polymers. Journal of Colloid and Interface Science. 2005;284:527-41.

15. Phenrat T, Saleh N, Sirk K, Tilton RD, Lowry GV. Aggregation and sedimentation of aqueous nanoscale zerovalent iron dispersions. Environmental Science & Technology. 2007;41:284-90.

16. Reindl A, Peukert W. Intrinsically stable dispersions of silicon nanoparticles. Journal of Colloid and Interface Science. 2008;325:173-8.

17. Wagner J, Fischer B. Field induced anisotropy of charged magnetic colloids: A rescaled mean spherical approximation study. The Journal of Chemical Physics. 2006;124:114901.

18. Leong SS, Ahmad Z, Low SC, Camacho J, Faraudo J, Lim J. Unified view of magnetic nanoparticle separation under magnetophoresis. Langmuir. 2020;36(28):8033-55.

19. Calderon FL, Stora T, Monval OM, Poulin P, Bibette J. Direct measurement of colloidal forces. Physical Review Letters. 1994;72:2959-62.

20. Montagne F, Braconnot S, Mondain-Monval O, Pichot C, Elaïssari A. Colloidal and physicochemical characterization of highly magnetic O/W magnetic emulsions. Journal of Dispersion Science and Technology 2003; 24, 821–832.

21. Shi Y-A, Ye M-P, Du L-C, Weng Y-X. Experimental determination of particle size-dependent surface charge density for silica nanospheres. Journal of Physical Chemistry 2018; 122, 23764–23771.
